# Supplementary material for: Counselling for Chronic Insomnia in Swiss Pharmacies: A Survey Study Based on Case Vignettes
Source: Pharmacy (Basel). 2023 Jun 16;11(3):105. doi: 10.3390/pharmacy11030105 (PMC10302654; doi:10.3390/pharmacy11030105)
Supplement: Supplementary file 1 [file pharmacy-11-00105-s001.zip › pharmacy-2423985-supplementary.pdf]

Supplementary Materials S1: Online questionnaire - English version

Variation in counselling clients* with chronic insomnia in Swiss pharmacies: a survey study using clinical vignettes.

Introduction text

Dear colleagues
Through this survey, we would like to describe how pharmacists in Switzerland counsel and treat adult clients (>18 years) with chronic insomnia. For this purpose, we present three clinical vignettes through which we would like to understand your current clinical activity. The clinical vignettes are followed by general questions about your perceptions of the prevalence of chronic insomnia and specifically about cognitive-behavioral therapy (CBT-I) for the treatment of chronic insomnia. We are also interested in your individual needs and suggestions that could help improve the treatment of clients with chronic insomnia. At the end of the survey, you will find a field for your free comments, that we encourage you to use.
The purpose of the questionnaire is not to test your knowledge, but rather to describe how clinical activity can sometimes differ from one pharmacy to another. Clinical activity is complex and most of the time, there is not a correct or wrong approach. As you know, clinical activity guidelines also differ. This work will help develop future interventions based on current clinical activity. The questionnaire was developed and tested interprofessionally with pharmacists, primary care physicians and specialists. According to our experience during the pre-tests, the questionnaire takes about 25 minutes to complete. All your answers will be collected anonymously. The data will be analysed afterwards and published in a scientific journal. Thank you for your participation.

1. Background information -
a. Personal data

Gender
❑ Man
❑ Woman
❑ Other

Age group (in years)
❑ <30
❑ 31-40
❑ 41-50
❑ 51-60
❑ >60

Since how long are your working in a community pharmacy
❑ <5 years
❑ 6-15 years
❑ 16-25 years
❑ 26-35 years
❑ >35 years

What is your function in the pharmacy?
❑ Business owner
❑ Administrator*in
❑ Day pharmacist*in
❑ Pharmacist* in a floater function
❑ Other: ____

At which university did you receive your master's degree or diploma in pharmacy?
❑ ETH Zürich
❑ University of Basel
❑ University of Geneva and/or Lausanne
❑ University of Bern
❑ Other university: ____

Have you done any additional training?
❑ Skills certificate "Anamnesis in primary care"
❑ Quality circle courses
❑ CAS "Clinical Pharmacy: Services in Primary Care" at the University of Geneva
❑ CAS "Clinical Pharmacy" at the University of Basel or at the ETH
❑ FPH in Community Pharmacy
❑ FPH in Hospital Pharmacy
❑ FPH in Clinical pharmacy
❑ Doctorate

❑ None
❑ Other: ________________________________

1. Background information
b. Data of the pharmacy

Location of the pharmacy
❑ Rural
❑ Agglomeration
❑ City centre

In which canton is your pharmacy located?
❑ Appenzell Ausserrhoden
❑ Appenzell Inner Rhodes
❑ Aargau
❑ Basel-Country
❑ Basel City
❑ Bern
❑ Freiburg
❑ Geneva
❑ Glarus
❑ Grisons
❑ Jura
❑ Lucerne
❑ Neuchâtel
❑ Nidwalden
❑ Obwalden
❑ St. Gallen
❑ Schaffhausen
❑ Schwyz
❑ Solothurn
❑ Ticino
❑ Thurgau
❑ Uri
❑ Vaud
❑ alais
❑ Zug
❑ Zurich

Which delivery modality are you subject to in your geographic sales area?
❑ Dispensing only at the pharmacy (Rx)
❑ Self-dispensing by physicians (SD)
❑ Mixed form (MF)

Pharmacy type
❑ Chain
❑ Grouping
❑ Purchasing group
❑ Independent pharmacy
❑ Other: ____

Number of employees in full-time equivalents
(Example a: 1 pharmacist 90% + 1 pharmacist 60% = 150 job percentages = 1.5 full-time equivalents)
Number of full-time pharmacists* (including students in their assistantship year): ____________full-time equivalents
(Example b: 1 pharmacy assistant 80% + 1 Pharmacy assistant in training 100% + 1 Chemist 70% = 250 job percentages = 2.5 full-time equivalents).
Number of employees involved in the dispensing of medicines (excluding pharmacists): _____________________________full-time equivalents

Clinical Vignette 1

Mrs. R. is a 50-year-old secretary in an international company. A few months ago, her company had to lay people off and she was under a lot of stress that kept her awake for several months. The situation has now stabilized, and she is confident that she can keep her job. However, her sleep has not improved. She has had trouble falling asleep for almost 4 months now. She also regularly wakes up during the night and then cannot get back to sleep. Her lack of sleep affects her work during the day, she feels less focused and has difficulty completing work that needs to be done in a timely manner. She comes to your pharmacy because she wants a medication that will relieve her of these sleep problems.

What over-the-counter pharmacologic or homeopathic treatments (including medications of the list B-/B+ - *prescription drugs dispensable by pharmacists with specific counselling and documentation*) would you consider for this client?

Tick the box that corresponds to your opinion

		0 (Does not apply at all)	1 (Does not apply)	2 (Does rather not apply)	3 (More likely to apply)	4 (Often applies)	5 (Strongly applies)	Don't know/No answer
Antihistamines (e.g. Diphenhydramine (Benocten®) or Doxylamine (Sanalepsi® N))		❑	❑	❑	❑	❑	❑	❑
Valerian (e.g. Sedonium®, Somnofor® or combined with other phytotherapeutics (Dormiplant®, Redormin®))		❑	❑	❑	❑	❑	❑	❑
Other phytotherapeutics (e.g. lavender (Lasea®), herbal tea)		❑	❑	❑	❑	❑	❑	❑
Homeopathic preparations		❑	❑	❑	❑	❑	❑	❑
Other treatments (mention below)		❑	❑	❑	❑	❑	❑	❑
None of the treatments mentioned here		❑	❑	❑	❑	❑	❑	❑

Other treatments you might consider: ____

In an interprofessional discussion, what prescription medications would you recommend to the physician or practitioner?

Tick the box that corresponds to your opinion

	0 (Does not apply at all)	1 (Does not apply)	2 (Does rather not apply)	3 (More likely to apply)	4 (Often applies)	5 (Strongly applies)	Don't know/No answer
Z-substances (e.g., Zolpidem (Stilnox®), Zopiclone (Imovane®))	❑	❑	❑	❑	❑	❑	❑
Benzodiazepines (e.g., Lorazepam (Seresta®))	❑	❑	❑	❑	❑	❑	❑
Antidepressants with sedative effects (e.g., Mirtazapine (Remeron®), Trazodone (Trittico®))	❑	❑	❑	❑	❑	❑	❑
Neuroleptics (e.g., Quetiapine (Sequase®, Seroquel®))	❑	❑	❑	❑	❑	❑	❑
Melatonin (e.g. Circadin®)	❑	❑	❑	❑	❑	❑	❑
Other prescription medications (mention below)	❑	❑	❑	❑	❑	❑	❑
Another medication but no recommendation of prescription drugs	❑	❑	❑	❑	❑	❑	❑

Other prescription medications you would recommend to the health care provider for this client:

____

What non-pharmacological treatments could you think of for this client?
Tick the box that corresponds to your opinion

	0 (Does not apply at all)	1 (Does not apply)	2 (Does rather not apply)	3 (More likely to apply)	4 (Often applies)	5 (Strongly applies)	Don't know/No answer
Sleep hygiene	❑	❑	❑	❑	❑	❑	❑
Bedtime/sleep restriction	❑	❑	❑	❑	❑	❑	❑
Cognitive Behavioral Therapy for Insomnia (CBT-I)	❑	❑	❑	❑	❑	❑	❑
Hypnosis	❑	❑	❑	❑	❑	❑	❑
Relaxation techniques	❑	❑	❑	❑	❑	❑	❑
Other psychotherapy	❑	❑	❑	❑	❑	❑	❑
Other non-pharmacological treatments (mention below)	❑	❑	❑	❑	❑	❑	❑
None of the non-pharmacological treatment mentioned here	❑	❑	❑	❑	❑	❑	❑

Other non-pharmacological treatments you might consider for this client: ___________________________________

Among the above options, what would be your FIRST CHOICE OF TREATMENT (pharmacologic (including medications of the list B+/B-), homeopathic, and/or non-pharmacologic) for this client at the pharmacy?
*You can specify a **first choice treatment** for pharmacological **OR** non-pharmacological treatment **OR** both.*
Non-prescription pharmacological or homeopathic treatments
❑ Antihistamines (e.g., Diphenhydramine (Benocten®) or Doxylamine (Sanalepsi® N)).
❑ Valerian (e.g. Sedonium®, Somnofor® or combined with other phytotherapeutics (Dormiplant®, Redormin®)).
❑ Other phytotherapeutics (lavender (Lasea®), herbal tea)
❑ Homeopathic preparations
❑ Other (mention below)
❑No over-the-counter pharmacological or homeopathic treatments.

AND/OR

Non-pharmacological treatment
❑ Sleep hygiene
❑ Bedtime/sleep restriction
❑ Cognitive Behavioral Therapy for Insomnia (CBT-I)
❑ Hypnosis
❑ Relaxation techniques
❑ Other psychotherapy
❑ Other (mention below)
❑ No non-pharmacological treatment
Other FIRST CHOICE OF TREATMENT: ____

What are the further clarifications you will discuss with your customer in such a situation?
Check the box that applies to you.

	Always or almost always (in ≥90% of cases)	Very often (in ≥50% - <90% of cases)	Frequently (in ≥10% - <50% of cases)	Rarely or never (in <10% of the cases)	Don't know/No answer
Delivery of a treatment (homeopathic or pharmacological (incl. B+/B-)) without "in-depth" anamnesis	❑	❑	❑	❑	❑
Sleep history	❑	❑	❑	❑	❑
Medicament history	❑	❑	❑	❑	❑
Review of comorbidities	❑	❑	❑	❑	❑
Personal history	❑	❑	❑	❑	❑

Anamnesis in own consulting room (instead of anamnesis at sales counter)	❑	❑	❑	❑	❑

Other elements you would clarify in the scope of the exchange with the patient:
____

Clinical Vignette 2A

Mrs. C. is a 45-year-old cashier with three children. She divorced less than a year ago and is now in financial difficulties. She regularly wakes up in the night and then can't get back to sleep each time. She feels tired all the time and finds it difficult to take care of the children when she gets home at night. A few months ago, she went to see a doctor and has been taking zolpidem every night for the past three months. The doctor renewed her prescription for zolpidem for another three months. This customer, that you see for the first time, comes to your pharmacy with the prescription to get this medication.

What over-the-counter pharmacologic or homeopathic treatments (including medication of the list B-/B+) might you consider for this client in addition to or as an alternative to zolpidem treatment?
Tick the box that corresponds to you

	0 (Does not apply at all)	1 (Does not apply)	2 (Does rather not apply)	3 (More likely to apply)	4 (Often applies)	5 (Strongly applies)	Don't know/No answer
Antihistamines (e.g. Diphenhydramine (Benocten®) or Doxylamine (Sanalepsi® N))	❑	❑	❑	❑	❑	❑	❑
Valerian (e.g. Sedonium®, Somnofor® or combined with other phytotherapeutics (Dormiplant®, Redormin®))	❑	❑	❑	❑	❑	❑	❑
Other phytotherapeutics (e.g. lavender (Lasea®), herbal tea)	❑	❑	❑	❑	❑	❑	❑
Homeopathic preparations	❑	❑	❑	❑	❑	❑	❑
Other treatments (mention below)	❑	❑	❑	❑	❑	❑	❑
None of the treatments mentioned here	❑	❑	❑	❑	❑	❑	❑

Other treatments you might consider for this client: ____

In an interprofessional discussion, what prescription medications would you recommend to the physician or practitioner as an alternative to Zolpidem?
Tick the box that corresponds to you

	0 (Does not apply at all)	1 (Does not apply)	2 (Does rather not apply)	3 (More likely to apply)	4 (Often applies)	5 (Strongly applies)	Don't know/No answer
No alternative: maintain Z-substances (e.g., Dolpidem (Stilnox®), Zopiclone (Imovane®))	❑	❑	❑	❑	❑	❑	❑
Benzodiazepines (e.g., Lorazepam (Seresta®))	❑	❑	❑	❑	❑	❑	❑
Antidepressants with sedative effects (e.g., Mirtazapine (Remeron®), trazodone (Trittico®))	❑	❑	❑	❑	❑	❑	❑
Neuroleptics (e.g., Quetiapine (Sequase®, Seroquel®))	❑	❑	❑	❑	❑	❑	❑
Melatonin (e.g. Circadin®)	❑	❑	❑	❑	❑	❑	❑
Other prescription medications (mention below).	❑	❑	❑	❑	❑	❑	❑
No recommendation of prescription drugs	❑	❑	❑	❑	❑	❑	❑

Other prescription medications you would recommend to the physician for this client: ______________________

What non-pharmacologic alternative treatments or in addition to Zolpidem could you think of for this client?
Tick the box that corresponds to you

	0 (Does not apply at all)	1 (Does not apply)	2 (Does rather not apply)	3 (More likely to apply)	4 (Often applies)	5 (Strongly applies)	Don't know/No answer
Sleep hygiene	❑	❑	❑	❑	❑	❑	❑
Bedtime/sleep restriction	❑	❑	❑	❑	❑	❑	❑
Cognitive Behavioral Therapy for Insomnia (CBT-I)	❑	❑	❑	❑	❑	❑	❑
Hypnosis	❑	❑	❑	❑	❑	❑	❑
Relaxation techniques	❑	❑	❑	❑	❑	❑	❑
Other psychotherapy	❑	❑	❑	❑	❑	❑	❑
Other non-pharmacological treatments (mention below)	❑	❑	❑	❑	❑	❑	❑
None of the non-pharmacological treatment mentioned here	❑	❑	❑	❑	❑	❑	❑

Other non-pharmacological treatments you might consider for this client: ______________________________

Among the above options, what would be your FIRST CHOICE OF TREATMENT (pharmacologic (including medications of the lists B+/B-), homeopathic, and/or non-pharmacologic) for this client at the pharmacy?
You can specify a ***first choice treatment*** *for pharmacological* ***OR*** *non-pharmacological treatment* ***OR*** *both.*
Non-prescription pharmacological or homeopathic treatments
❑ Antihistamines (e.g., Diphenhydramine (Benocten®) or Doxylamine (Sanalepsi® N)).
❑ Valerian (e.g. Sedonium®, Somnofor® or combined with other phytotherapeutics (Dormiplant®, Redormin®)).
❑ Other phytotherapeutics (lavender (Lasea®), herbal tea)
❑ Homeopathic preparations
❑ Other (mention below)
❑ No over-the-counter pharmacological or homeopathic treatments.

AND/OR

Non-pharmacological treatment
❑ Sleep hygiene
❑ Bedtime/sleep restriction
❑ Cognitive Behavioral Therapy for Insomnia (CBT-I)
❑ Hypnosis
❑ Relaxation techniques
❑ Other psychotherapy
❑ Other (mention below)
❑ No non-pharmacological treatment
Other **FIRST CHOICE OF TREATMENT**: ______________________________

Clinical Vignette 2B

Mr. G. is a 79-year-old man who is in a similar situation as Ms. C. in Clinical Vignette 2A, he wakes up several times during the night and has problems falling asleep. Because of his sleep problems, he is often tired during the day and cannot do all the tasks he wanted to do. He receives the same medical treatment (Zolpidem every night for 3 months) as Ms. C to improve his sleep.

What over-the-counter pharmacologic or homeopathic treatments (including medications of the lists B-/B+) could you think of for this client in addition to or as an alternative to zolpidem treatment?
Tick the box that corresponds to you

	0 (Does not apply at all)	1 (Does not apply)	2 (Does rather not apply)	3 (More likely to apply)	4 (Often applies)	5 (Strongly applies)	Don't know/No answer
Antihistamines (e.g. Diphenhydramine (Benocten®) or Doxylamine (Sanalepsi® N))	❑	❑	❑	❑	❑	❑	❑
Valerian (e.g. Sedonium®, Somnofor® or combined with other phytotherapeutics (Dormiplant®, Redormin®))	❑	❑	❑	❑	❑	❑	❑
Other phytotherapeutics (e.g. lavender (Lasea®), herbal tea).	❑	❑	❑	❑	❑	❑	❑

Homeopathic preparations	❑	❑	❑	❑	❑	❑	❑
Other treatments (mention below)	❑	❑	❑	❑	❑	❑	❑
None of the treatments mentioned here	❑	❑	❑	❑	❑	❑	❑

Other treatments you might consider for this client: ____

In an interprofessional discussion, what prescription medications would you recommend to the physician or practitioner as an alternative to Zolpidem?

Tick the box that corresponds to you

	0 (Does not apply at all)	1 (Does not apply)	2 (Does rather not apply)	3 (More likely to apply)	4 (Often applies)	5 (Strongly applies)	Don't know/No answer
No alternative: maintain Z-substances (e.g., Zolpidem (Stilnox®), Zopiclone (Imovane®))	❑	❑	❑	❑	❑	❑	❑
Benzodiazepines (e.g., Lorazepam (Seresta®))	❑	❑	❑	❑	❑	❑	❑
Antidepressants with sedative effects (e.g., Mirtazapine (Remeron®), Trazodone (Trittico®))	❑	❑	❑	❑	❑	❑	❑
Neuroleptics (e.g., Quetiapine (Sequase®, Seroquel®))	❑	❑	❑	❑	❑	❑	❑
Melatonin (e.g. Circadin®)	❑	❑	❑	❑	❑	❑	❑
Other prescription medications (mention below).	❑	❑	❑	❑	❑	❑	❑
No recommendation of prescription drugs	❑	❑	❑	❑	❑	❑	❑

Other prescription medications you would recommend to the doctor*s office for this client: ____________________________

What non-pharmacologic alternative treatments or in addition to zolpidem could you think of for this client?

Tick the box that corresponds to you

	0 (Does not apply at all)	1 (Does not apply)	2 (Does rather not apply)	3 (More likely to apply)	4 (Often applies)	5 (Strongly applies)	Don't know/No answer
Sleep hygiene	❑	❑	❑	❑	❑	❑	❑
Bedtime/sleep restriction	❑	❑	❑	❑	❑	❑	❑
Cognitive Behavioral Therapy for Insomnia (CBT-I)	❑	❑	❑	❑	❑	❑	❑
Hypnosis	❑	❑	❑	❑	❑	❑	❑
Relaxation techniques	❑	❑	❑	❑	❑	❑	❑
Other psychotherapy	❑	❑	❑	❑	❑	❑	❑
Other non-pharmacological treatments (mention below)	❑	❑	❑	❑	❑	❑	❑
None of the non-pharmacological treatment mentioned here	❑	❑	❑	❑	❑	❑	❑

Other non-pharmacological treatments you might consider for this client: __________________________________

Among the above options, what would be your FIRST CHOICE OF TREATMENT (pharmacologic (including medications of the lists B+/B-), homeopathic, and/or non-pharmacologic) for this client at the pharmacy?

You can specify a ***first choice treatment*** *for pharmacological* ***OR*** *non-pharmacological treatment* ***OR*** *both.*

Non-prescription pharmacological or homeopathic treatments

❑ Antihistamines (e.g., Diphenhydramine (Benocten®) or Foxylamine (Sanalepsi® N)).
❑ Valerian (e.g. Sedonium®, Somnofor® or combined with other phytotherapeutics (Dormiplant®, Redormin®)).
❑ Other phytotherapeutics (e.g., lavender (Lasea®), herbal tea)
❑ Homeopathic preparations
❑ Other (mention below)
❑ No over-the-counter pharmacological or homeopathic treatments

AND/OR

Non-pharmacological treatment
❑ Sleep hygiene
❑ Bedtime/sleep restriction
❑ Cognitive Behavioral Therapy for Insomnia (CBT-I)
❑ Hypnosis
❑ Relaxation techniques
❑ Other psychotherapy
❑ Other (mention below)
❑ No non-pharmacological treatment
Other FIRST CHOICE OF TREATMENT: ____

3. General perception of insomnia

In your opinion, how high is the percentage of customers who suffer from sleep disorders?
Regarding any cause
Subjective evaluation
❑ <5%
❑ 5%-15%
❑ 15%-30%
❑ 30-50%
❑ 50%-75%
❑ >75%
❑ Don't know/no answer

In your opinion, what percentage of clients suffer from acute insomnia (3 or more nights per week of insomnia for LESS than 3 months)?
Acute insomnia was not necessarily previously diagnosed
Subjective assessment of the ratio
❑ <5%
❑ 5%-15%
❑ 15%-30%
❑ 30-50%
❑ 50%-75%
❑ >75%
❑ Don't know/no answer

In your opinion, what percentage of clients suffer from chronic insomnia (3 or more nights per week of insomnia for MORE than 3 months)?
Chronic insomnia was not necessarily previously diagnosed
Subjective assessment of the ratio
❑ <5%
❑ 5%-15%
❑ 15%-30%
❑ 30-50%
❑ 50%-75%
❑ >75%
❑ Don't know/no answer

In your opinion, what is the proportion of clients who take benzodiazepines or the Z-substance regularly and long-term (>1x/week for >6 months)?
All prescription indications together
Subjective rating
❑ <5%
❑ 5%-15%
❑ 15%-30%
❑ 30-50%
❑ 50%-75%
❑ >75%
❑ Don't know/no answer

How would you rate the effectiveness of the following homeopathic and pharmacologic treatments (pharmacologic treatments with or without a physician's prescription) in addition to the placebo effect in the short-term (<1 month) treatment of chronic insomnia (3 or more nights per week of insomnia for MORE than 3 months)?
Tick the box that corresponds to you

	0 (Not effective at all)	1 (Not effective)	2 (Rather not effective)	3 (Rather effective)	4 (Very effective)	5 (Extremely effective)	Don't know/No answer
Antihistamines (e.g. Diphenhydramine (Benocten®) or Doxylamine (Sanalepsi® N))	❑	❑	❑	❑	❑	❑	❑
Valerian (e.g. Sedonium®, Somnofor® or combined with other phytotherapeutics (Dormiplant®, Redormin®))	❑	❑	❑	❑	❑	❑	❑
Other phytotherapeutics (e.g. lavender (Lasea®), herbal tea)	❑	❑	❑	❑	❑	❑	❑
Homeopathic preparations	❑	❑	❑	❑	❑	❑	❑
Z-substance (e.g. Zolpidem (Stilnox®), Zopiclone (Imovane®))	❑	❑	❑	❑	❑	❑	❑
Benzodiazepines (e.g., Lorazepam (Seresta®))	❑	❑	❑	❑	❑	❑	❑
Antidepressants with sedative effects (e.g., Mirtazapine (Remeron®), Trazodone (Trittico®))	❑	❑	❑	❑	❑	❑	❑
Neuroleptics (e.g., Quetiapine (Sequase®, Seroquel®))	❑	❑	❑	❑	❑	❑	❑
Melatonin (e.g. Circadin®)	❑	❑	❑	❑	❑	❑	❑

How often do you recommend the following elements of sleep hygiene to your clients?

	Always or almost always (in ≥90% of cases)	Very often (in ≥50% - <90% of cases)	Frequently (in ≥10% - <50% of cases)	Rarely or never (in <10% of the cases)	Don't know/ No answer
No of cups of coffee, tea or cocoa after 4:00 p.m.	❑	❑	❑	❑	○
Light dinner	❑	❑	❑	❑	○
Physical exercise during the day, but not in the evening	❑	❑	❑	❑	○
No screen time right before bed	❑	❑	❑	❑	○
Introduce ritual at bedtime	❑	❑	❑	❑	○
Cools the temperature of the bedroom	❑	❑	❑	❑	○
Relaxation method	❑	❑	❑	❑	○
Light therapy	❑	❑	❑	❑	○
Do not stay in bed with insomnia	❑	❑	❑	❑	○
Bedtime/sleep restriction	❑	❑	❑	❑	○
Wake up always at the same time	❑	❑	❑	❑	○
Avoid naps during the day	❑	❑	❑	❑	○

Other: ____

On average, how much time do you need to talk to one patient about sleep disorders (history and treatment options)?

❑ <5 min
❑ 5-10 min
❑ 10-15 min
❑ >15 min

❑ Don't know/no answer

On average, how much time do you need to counsel one patient about sleep hygiene?
❑ <5 min
❑ 5-10 min
❑ 10-15 min
❑ >15 min
❑ Don't know/no answer

Please think about the last 10 clients you counselled for chronic insomnia. How many of their counselling sessions did you do in your counselling room?
❑ None
❑ 1-2 times
❑ 3-5 times
❑ 6-7 times
❑ 8-9 times
❑ All
❑ Don't know/no answer

Do you have an algorithm in your pharmacy for treating customers with sleep disorders or chronic insomnia?
❑ Yes
❑ No
❑ Don't know/no answer

We would be very grateful if you could upload the algorithm or send it to the following e-mail address: fanny.mulder@students.unibe.ch
The address can also be found in the e-mail.

Would you be interested in having an algorithm at your disposal for treating sleep disorders and chronic insomnia?
❑ Yes
❑ No
❑ Don't know/No answer
❑ Comments

4. CBT-I knowledge and experience

According to new guidelines for the treatment of chronic insomnia, cognitive behavioral therapy for insomnia (CBT-I) is the first-line treatment[1] . CBT-I is a behavioral therapy that includes psychoeducation, relaxation techniques, various cognitive methods, sleep restriction, and stimulus control. While CBT-I has been offered mainly by specialized psychologists, nowadays an alternative online form exists and could reach a larger part of the population.
[1]i.m@ail-offizin, Herbal Sedatives and Hypnotics, No. 11 / 15.06.2020

Before filling out this questionnaire, have you ever heard of CBT-I?
❑ Yes
❑ No
❑ Don't know/no answer
❑ Comments

If you have heard of CBT-I, have you ever recommended CBT-I to your clients?
❑ Never
❑ <1x/month
❑ 1x/month to <1x/week
❑ 1x/week or more
❑ Don't know/no answer

Do you know a psychologist or CBT-I specialist you could recommend to your clients?
❑ Yes
❑ No
❑ Don't know/no answer

Have you recommended clients to see a psychologist or CBT-I specialist in the past?
❑ Never
❑ <1x/month
❑ 1x/month to <1x/week
❑ 1x/week or more
❑ Don't know/no answer

Do you know of a CBT-I website/app that you could recommend to your clients?
❑ Yes
❑ No
❑ Don't know/no answer

Have you advised clients on a CBT-I website/application in the past?
❑ Never
❑ <1x/month
❑ 1x/month to <1x/week
❑ 1x/week or more
❑ Don't know/no answer

How interested are you in following clients online for several weeks after CBT-I therapy in the future to help them complete therapy?
❑ None
❑ A little
❑ Medium
❑ Much
❑ Very much
❑ Don't know/no answer
❑ Comments/notes/ideas

How interested are you in further training/courses on sleep disorders and CBT-I?
❑ None
❑ A little
❑ Medium
❑ Much
❑ Very much
❑ Don't know/no answer
❑ Comments/notes/ideas

In your opinion, what else would you need to implement a good insomnia treatment for your customers in your pharmacy?
❑ More knowledge/courses on the subject
❑ More space to have a personal conversation with clients (e.g., separate treatment room)
❑ The possibility to charge for the service of insomnia counseling (e.g., CBT-I)
❑ Better contact with doctors and physicians
❑ Other

Other questions/comments/suggestions/ideas for sleep disorder treatment.
____

Supplementary Materials S2: Detailed results of the pharmacists' recommendation for the three case vignettes

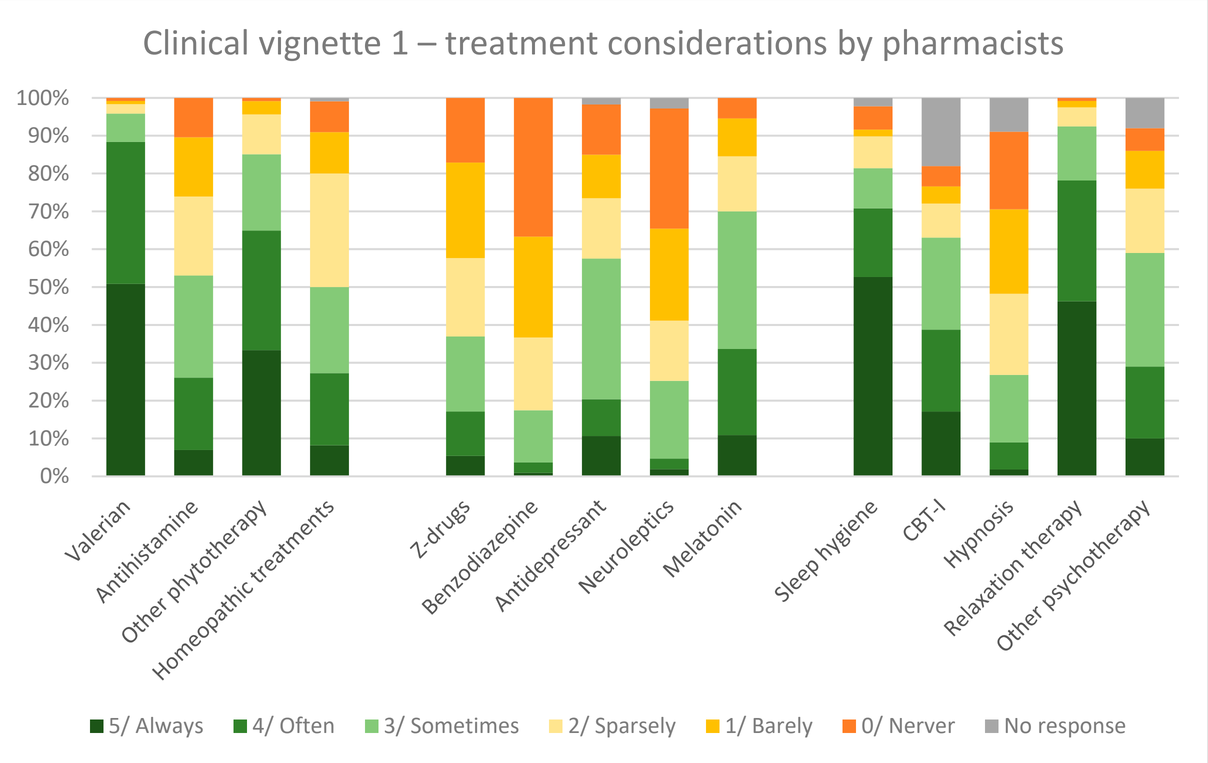

Figure S1 describes which treatments the participants would consider for the client of the first clinical vignette, a 50 years old secretary without prescription. The treatment options were separated into three categories, the first one being pharmacological and homeopathic treatments without prescription (including medication of the lists B-/B+, prescription medications dispensable by pharmacists with appropriate counselling and documentation), the second one being medication with prescription they would recommend to a primary care provider during an interprofessional discussion and the third category encompassing non-pharmacological treatments.

The treatment that the participants considered preferentially for this client was valerian (96%, n=115/120), closely followed by relaxation therapy (92%, n=110/119).

However, less than two-thirds of the participants would recommend CBT-I (63%, n=70/110). In general, the medication that needs a prescription were less often recommended with about a third considering Z-drugs (37%, n=41/111), a quarter in favour of neuroleptics (25%, n=27/107) and only a few would consider a benzodiazepine (17%, n=19/109).

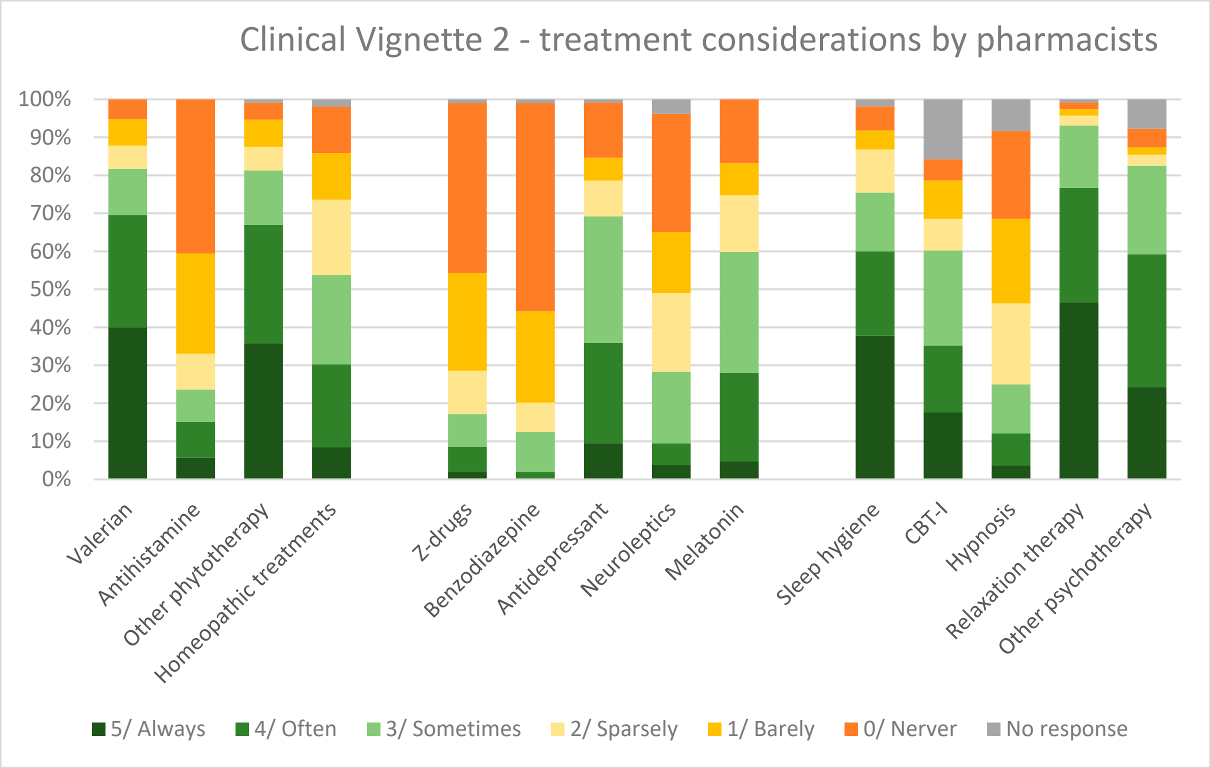

Figure S2 describes which treatments the participants would consider for the client of the second clinical vignette, a 45 year old cashier with a renewed prescription for three more months of zolpidem. The treatment options are the same as depicted in figure S1, separated into the same categories.

Here, the treatment that the Swiss pharmacists considered preferentially for this client was relaxation therapy (93%, n=108/116) followed by other psychotherapy options (83%, n=85/103).

However, CBT-I was recommended by less than two-thirds of the participants (60%, n=65/108). Comparable to the patient of clinical vignette 1, medications needing a prescription were less often recommended with even fever recommending Z-drugs (e.g., Zolpidem) 18%, n=18/105) or considering a benzodiazepine (13%, n=13/104).

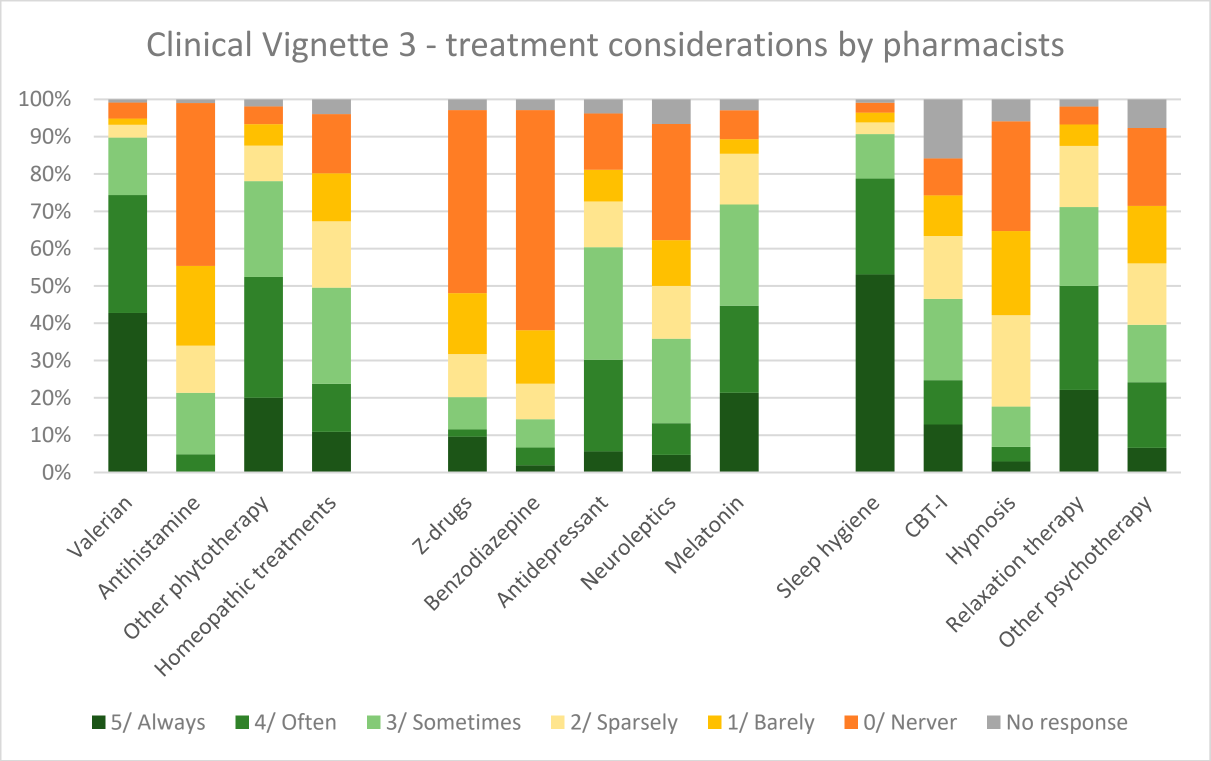

Figure S3 describes which treatments the participants would consider for the client of the second clinical vignette, the 79 years old man that had otherwise similar characteristics to the clients of the second vignettes. The treatment options and the categories are the same as in vignette 1 and 2.

The treatment that the pharmacists considered preferentially for this client was sleep hygiene (91%, n=103/113) followed by valerian (90%, n=105/117). As for CBT-I, it was recommended by less than half of the participants (47%, n=47/101). Here too, medications needing a prescription were less often recommended with 20% of pharmacists (n=21/104) recommending Z-drugs or 14% (n=15/105) considering a benzodiazepine.

Supplementary Materials S3: Online questionnaire, German online version

Fragebogen: Unterschiede in der Beratung von Kund*innen mit chronischer Insomnie in Schweizer Apotheken: eine Befragungsstudie anhand von klinischen Vignetten

Einführungstext

Sehr geehrte Kolleginnen und Kollegen
Anhand dieser Umfrage möchten wir beschreiben, wie Apotheker*innen in der Schweiz erwachsene Kund*innen (>18 Jahre) mit chronischer Insomnie beraten und behandeln. Zu diesem Zweck stellen wir Ihnen drei klinische Vignetten vor, durch die wir Ihre aktuelle klinische Tätigkeit verstehen möchten. Auf die klinischen Vignetten folgen allgemeine Fragen zu Ihrer Wahrnehmung von der Prävalenz von chronischer Insomnie und speziell zu kognitiver Verhaltenstherapie zur Behandlung von chronischer Insomnie (KVT-I oder auf Englisch: cognitive-behanvioral therapy for insonmia CBT-I). Wir sind auch an Ihren individuellen Bedürfnissen und Vorschlägen interessiert, die helfen könnten, die Behandlung von Kund*innen mit chronischer Insomnie zu verbessern. Am Ende der Umfrage finden Sie ein Feld für freie Kommentare, welches Sie nutzen können.
Der Zweck des Fragebogens ist nicht, Ihr Wissen zu testen, sondern vielmehr zu beschreiben, wie sich die klinische Tätigkeit manchmal von einer Apotheke zur anderen unterscheiden kann. Die klinische Tätigkeit ist komplex und es gibt nicht immer einen richtigen oder falschen Ansatz. Wie Sie wissen, unterscheiden sich auch die Richtlinien für die klinische Tätigkeit. Diese Arbeit wird dazu beitragen, zukünftige Interventionen auf der Grundlage der aktuellen klinischen Tätigkeit zu entwickeln.
Der Fragebogen wurde interprofessionell und mit Apothekern bzw. Apothekerinnen und Ärzten der Primärversorgung entwickelt und getestet. Nach unseren Erfahrungen im Rahmen der Vorversuche dauert das Ausfüllen des Fragebogens bei den Vorversuchen ca. 25 Minuten. Alle Ihre Antworten werden anonymisiert gesammelt Die Daten werden anschliessend analysiert und in einer Fachzeitschrift veröffentlicht. Vielen Dank für Ihre Teilnahme.

1. Hintergrundinformationen - a. Personenbezogene Daten

Geschlecht

◯ Mann
◯ Frau
◯ Anderes

Altersgruppe (Angabe in Jahren)

◯ <30
◯ 31-40
◯ 41-50
◯ 51-60
◯ >60

Berufserfahrung in einer öffentlichen Apotheke (Angabe in Jahren)

◯ <5
◯ 6-15
◯ 16-25
◯ 26-35
◯ >35

Was ist Ihre Funktion in der Apotheke?

◯ Geschäftsinhaber*in
◯ Verwalter*in
◯ Tagesapotheker*in
◯ Apotheker*in in Springerfunktion
◯ Anderes: ____________________

An welcher Universität haben Sie Ihren Master-Abschluss resp. Ihr Diplom in Pharmazie erhalten?

◯ ETH Zürich
◯ Universität Basel
◯ Universität Genf und/oder Lausanne
◯ Universität Bern
◯ Andere Universität: ____________________

Haben Sie eine Zusatzausbildung gemacht?

☐ Fähigkeitsausweis «Anamnese in der Grundversorgung»
☐ Qualitätszirkel-Kurse
☐ CAS "Klinische Pharmazie: Dienstleistungen in der Grundversorgung" an der Universität Genf
☐ CAS "Klinische Pharmazie" an der Universität Basel oder an der ETH
☐ FPH Offizinpharmazie
☐ FPH in Spitalpharmazie

☐ FPH in klinischer Pharmazie
☐ Doktorat
☐ Keine
☐ Andere: ______________________________

1. Hintergrundinformationen - b. Daten der Apotheke

Lage der Apotheke
◯ Ländlich
◯ Agglomeration
◯ Stadtzentrum

In welchem Kanton befindet sich Ihre Apotheke?
◯ Appenzell Ausserrhoden
◯ Appenzell Innerrhoden
◯ Aargau
◯ Basel-Landschaft
◯ Basel-Stadt
◯ Bern
◯ Freiburg
◯ Genf
◯ Glarus
◯ Graubünden
◯ Jura
◯ Luzern
◯ Neuenburg
◯ Nidwalden
◯ Obwalden
◯ St. Gallen
◯ Schaffhausen
◯ Schwyz
◯ Solothurn
◯ Tessin
◯ Thurgau
◯ Uri
◯ Waadt
◯ Wallis
◯ Zug
◯ Zürich

Welcher Abgabemodalität unterliegen Sie in Ihrem geografischen Verkaufsgebiet?
◯ Abgabe nur in der Apotheke (Rx)
◯ Selbstdispensation durch Ärzte bzw. Ärztinnen (SD)
◯ Mischform (MF)

Typ der Apotheke
◯ Kette
◯ Gruppierung
◯ Einkaufsgemeinschaft
◯ Unabhängige Apotheke
◯ Andere

Anzahl Mitarbeitenden in Vollzeitäquivalenten
(Beispiel a: 1 Apotheker 90% + 1 Apothekerin 60% = 150 Stellenprozente = 1,5 Vollzeitstellen)
Anzahl vollzeitbeschäftigter Apotheker*innen (einschließlich Studenten im Assistentenjahr): ___________Vollzeitstellen
(Beispiel b: 1 Pharma-Assistentin 80% + 1 PA in Ausbildung 100% + 1 Drogist 70% = 250 Stellenprozente = 2,5 Vollzeitstellen)
Anzahl Mitarbeiter/innen, welche bei der Abgabe von Medikamenten mitwirken (mit Ausnahme von Apotheker*innen):
__________________________Vollzeitstellen

2. Klinische Vignette - Klinische Vignette 1

Frau R. ist eine 50-jährige Sekretärin in einer internationalen Firma. Vor ein paar Monaten musste ihre Firma Leute entlassen und sie stand unter großem Stress, der sie mehrere Monate lang nicht schlafen liess. Die Situation hat sich nun stabilisiert und sie ist zuversichtlich, dass sie ihren Job behalten kann. Ihr Schlaf hat sich jedoch nicht verbessert. Seit fast 4 Monaten hat sie Probleme beim Einschlafen. Außerdem wacht sie regelmäßig in der Nacht auf und kann dann nicht mehr einschlafen. Ihr Schlafmangel wirkt sich auf ihre Arbeit während des Tages aus, sie fühlt sich weniger konzentriert und hat Schwierigkeiten, die anfallenden Arbeiten

rechtzeitig zu erledigen. Sie kommt in Ihre Apotheke, weil sie ein Medikament möchte, welches sie von diesen Schlafproblemen befreit.

Welche rezeptfreien pharmakologischen oder homöopathischen Behandlungen (inkl. Medikamente der Liste B-/B+) könnten Sie sich für diese Kundin vorstellen?

Kreuzen Sie das Kästchen an, das Ihnen entspricht

	0 (Trifft überhaupt nicht zu)	1 (Trifft nicht zu)	2 (Trifft eher nicht zu)	3 (Trifft eher zu)	4 (Trifft oft zu)	5 (Trifft ganz zu)	Weiß nicht/Keine Antwort
Antihistaminika (z.B. Diphenhydramin (Benocten®) oder Doxylamin (Sanalepsi® N))	○	○	○	○	○	○	○
Baldrian (z.B. Sedonium®, Somnofor® oder kombiniert mit anderen Phytotherapeutika (Dormiplant®, Redormin®))	○	○	○	○	○	○	○
Andere Phytotherapeutika (z.B. Lavendel (Lasea®), Kräutertee)	○	○	○	○	○	○	○
Homöopathische Präparate	○	○	○	○	○	○	○
Andere Behandlungen (weiter unten erwähnen)	○	○	○	○	○	○	○
Keine der hier genannten Behandlungen	○	○	○	○	○	○	○

Andere Behandlungen, die Sie sich vorstellen könnten:

____

Welche verschreibungspflichtigen Medikamente würden Sie dem Arzt bzw. der Ärztin in einer interprofessionellen Diskussion empfehlen?

Kreuzen Sie das Kästchen an, das Ihnen entspricht

	0 (Trifft überhaupt nicht zu)	1 (Trifft nicht zu)	2 (Trifft eher nicht zu)	3 (Trifft eher zu)	4 (Trifft oft zu)	5 (Trifft ganz zu)	Weiß nicht/Keine Antwort
Z-Substanzen (z.B. Zolpidem (Stilnox®), Zopiclon (Imovane®))	○	○	○	○	○	○	○
Benzodiazepine (z.B. Lorazepam (Seresta®))	○	○	○	○	○	○	○
Antidepressiva mit sedierender Wirkung (z.B. Mirtazapin (Remeron®), Trazodon (Trittico®))	○	○	○	○	○	○	○
Neuroleptika (z.B. Quetiapin (Sequase®, Seroquel®))	○	○	○	○	○	○	○
Melatonin (z.B. Circadin®)	○	○	○	○	○	○	○
Andere verschreibungspflichtige Medikamente (weiter unten erwähnen)	○	○	○	○	○	○	○
Keine Empfehlung von verschreibungspflichtigen Medikamenten	○	○	○	○	○	○	○

Andere verschreibungspflichtige Medikamente, die Sie dem Arzt bzw. der Ärztin für diese Kundin empfehlen würden:

____

Welche nicht-pharmakologischen Behandlungen könnten Sie sich für diese Kundin vorstellen?

(Kreuzen Sie das Kästchen an, das Ihnen entspricht

	0 (Trifft überhaupt nicht zu)	1 (Trifft nicht zu)	2 (Trifft eher nicht zu)	3 (Trifft eher zu)	4 (Trifft oft zu)	5 (Trifft ganz zu)	Weiß nicht/Keine Antwort
Schlafhygiene	○	○	○	○	○	○	○
Bettzeit-/Schlafrestriktion	○	○	○	○	○	○	○
Kognitive Verhaltenstherapie für Insomnie (KVT-I/CBT-I)	○	○	○	○	○	○	○
Hypnose	○	○	○	○	○	○	○
Entspannungstechniken	○	○	○	○	○	○	○

Andere Psychotherapie	◯	◯	◯	◯	◯	◯	◯
Andere nicht-pharmakologische Behandlungen (weiter unten erwähnen)	◯	◯	◯	◯	◯	◯	◯
Keine der hier genannten nicht-pharmakologische Behandlung	◯	◯	◯	◯	◯	◯	◯

Andere nicht-pharmakologische Behandlungen, die Sie sich für diese Kundin vorstellen könnten:
____

Unter den oben genannten Optionen, was wäre für Sie die ERSTE BEHANDLUNGSWAHL (pharmakologische (inkl. Liste B+/B-), homöopathische und/oder nicht-pharmakologische) für diese Kundin in der Apotheke?
Sie können eine ***Behandlung erster Wahl*** *für eine pharmakologische* ***ODER*** *nicht-pharmakologische Behandlung* ***ODER*** *beides angeben.*
Rezeptfreie pharmakologische oder homöopathische Behandlungen
◯ Antihistaminika (z.B. Diphenhydramin (Benocten®) oder Doxylamin (Sanalepsi® N))
◯ Baldrian (z.B. Sedonium®, Somnofor® oder kombiniert mit anderen Phytotherapeutika (Dormiplant®, Redormin®))
◯ Andere Phytotherapeutika (Lavendel (Lasea®), Kräutertee)
◯ Homöopathische Präparate
◯ Andere (weiter unten erwähnen)
◯ Keine rezeptfreie pharmakologische oder homöopathische Behandlungen

UND/ODER

Nicht-pharmakologische Behandlung
◯ Schlafhygiene
◯ Bettzeit-/Schlafrestriktion
◯ Kognitive Verhaltenstherapie für Insomnie (KVT-I/CBT-I)
◯ Hypnose
◯ Entspannungstechniken
◯ Andere Psychotherapie
◯ Andere (weiter unten erwähnen)
◯ Keine nicht-pharmakologische Behandlung
Sonstige ERSTE BEHANDLUNGSWAHL: ____

Was sind die weiteren Abklärungen, die Sie in einer solchen Situation mit Ihrer Kundin bzw. Ihrem Kunden besprechen werden?
Markieren Sie das Kästchen, das auf Sie zutrifft.

	Immer oder fast immer (in ≥90% der Gespräche)	Sehr oft (in ≥50% - <90% der Gespräche)	Häufig (in ≥10% - <50% der Gespräche)	Selten oder nie (in <10% der Gespräche)	Weiß nicht/Keine Antwort
Abgabe eine Behandlung (homöopathische oder pharmakologische (inkl. B+/B-)) ohne «eingehende» Anamnese	◯	◯	◯	◯	◯
Schlaf-Anamnese	◯	◯	◯	◯	◯
Arzneimittel Anamnese	◯	◯	◯	◯	◯
Überprüfung der Komorbiditäten	◯	◯	◯	◯	◯
Persönliche Geschichte	◯	◯	◯	◯	◯
Anamnese im eigenen Beratungsraum (statt Anamnese an Verkaufstheke)	◯	◯	◯	◯	◯

Andere Elemente der Abklärung: ____

2. Klinische Vignette - Klinische Vignette 2A

Frau C. ist eine 45-jährige Kassiererin mit drei Kindern. Sie ließ sich vor weniger als einem Jahr scheiden und steckt nun in finanziellen Schwierigkeiten. Sie wacht regelmässig in der Nacht auf und kann dann jeweils nicht mehr einschlafen. Sie fühlt sich ständig müde und findet es schwierig, sich um die Kinder zu kümmern, wenn sie abends nach Hause kommt. Vor einigen Monaten ging sie zu einer Ärztin und nimmt seit drei Monaten jeden Abend Zolpidem. Die Ärztin verlängerte ihr Rezept für Zolpidem für weitere drei Monate. Diese Kundin, die Sie zum ersten Mal sehen, kommt mit dem Rezept in Ihre Apotheke um das Medikament zu

beziehen.

Welche rezeptfreien pharmakologischen oder homöopathischen Behandlungen (inkl. Medikamente der Liste B-/B+) könnten Sie sich für diese Kundin vorstellen zusätzlich oder als Alternative zur Zolpidem-Behandlung?
Kreuzen Sie das Kästchen an, das Ihnen entspricht

	0 (Trifft überhaupt nicht zu)	1 (Trifft nicht zu)	2 (Trifft eher nicht zu)	3 (Trifft eher zu)	4 (Trifft oft zu)	5 (Trifft ganz zu)	Weiß nicht/Keine Antwort
Antihistaminika (z.B. Diphenhydramin (Benocten®) oder Doxylamin (Sanalepsi® N))	○	○	○	○	○	○	○
Baldrian (z.B. Sedonium®, Somnofor® oder kombiniert mit anderen Phytotherapeutika (Dormiplant®, Redormin®))	○	○	○	○	○	○	○
Andere Phytotherapeutika (z.B. Lavendel (Lasea®), Kräutertee)	○	○	○	○	○	○	○
Homöopathische Präparate	○	○	○	○	○	○	○
Andere Behandlungen (weiter unten erwähnen)	○	○	○	○	○	○	○
Keine der hier genannten Behandlungen	○	○	○	○	○	○	○

Andere Behandlungen, die Sie sich für diese Kundin vorstellen könnten:
____

Welche verschreibungspflichtigen Medikamente würden Sie dem Arzt bzw. der Ärztin in einer interprofessionellen Diskussion empfehlen als Alternative zu Zolpidem?
Kreuzen Sie das Kästchen an, das Ihnen entspricht

	0 (Trifft überhaupt nicht zu)	1 (Trifft nicht zu)	2 (Trifft eher nicht zu)	3 (Trifft eher zu)	4 (Trifft oft zu)	5 (Trifft ganz zu)	Weiß nicht/Keine Antwort
Keine Alternative: Z-Substanzen beibehalten (z.B. Zolpidem (Stilnox®), Zopiclon (Imovane®))	○	○	○	○	○	○	○
Benzodiazepine (z.B. Lorazepam (Seresta®))	○	○	○	○	○	○	○
Antidepressiva mit sedierender Wirkung (z.B. Mirtazapin (Remeron®), Trazodon (Trittico®))	○	○	○	○	○	○	○
Neuroleptika (z.B. Quetiapin (Sequase®, Seroquel®))	○	○	○	○	○	○	○
Melatonin (z.B. Circadin®)	○	○	○	○	○	○	○
Andere verschreibungspflichtige Medikamente (weiter unten erwähnen)	○	○	○	○	○	○	○
Keine Empfehlung von verschreibungspflichtigen Medikamenten	○	○	○	○	○	○	○

Andere verschreibungspflichtige Medikamente, die Sie dem Arzt*in für diese Kundin empfehlen würden:
____

Welche nicht-pharmakologischen Alternativbehandlungen oder zusätzlich zu Zolpidem könnten Sie sich für diese Kundin vorstellen?
Kreuzen Sie das Kästchen an, das Ihnen entspricht

	0 (Trifft überhaupt nicht zu)	1 (Trifft nicht zu)	2 (Trifft eher nicht zu)	3 (Trifft eher zu)	4 (Trifft oft zu)	5 (Trifft ganz zu)	Weiß nicht/Keine Antwort
Schlafhygiene	○	○	○	○	○	○	○

Bettzeit-/Schlafrestriktion	○	○	○	○	○	○	○
Kognitive Verhaltenstherapie für Insomnie (KVT-I/CBT-I)	○	○	○	○	○	○	○
Hypnose	○	○	○	○	○	○	○
Entspannungstechniken	○	○	○	○	○	○	○
Andere Psychotherapie	○	○	○	○	○	○	○
Andere nicht-pharmakologische Behandlungen (weiter unten erwähnen)	○	○	○	○	○	○	○
Keine der hier genannten nicht-pharmakologisch Behandlung	○	○	○	○	○	○	○

Andere nicht-pharmakologische Behandlungen, die Sie sich für diese Kundin vorstellen könnten:
____

Unter den oben genannten Optionen, was wäre für Sie die ERSTE BEHANDLUNGSWAHL (pharmakologische (inkl. Liste B+/B-), homöopathische und/oder nicht-pharmakologische) für diese Kundin in der Apotheke?
*Sie können eine **Behandlung erster Wahl** für eine pharmakologische **ODER** nicht-pharmakologische Behandlung **ODER** beides angeben.*
Rezeptfreie pharmakologische oder homöopathische Behandlungen
○ Antihistaminika (z.B. Diphenhydramin (Benocten®) oder Doxylamin (Sanalepsi® N))
○ Baldrian (z.B. Sedonium®, Somnofor® oder kombiniert mit anderen Phytotherapeutika (Dormiplant®, Redormin®))
○ Andere Phytotherapeutika (Lavendel (Lasea®), Kräutertee)
○ Homöopathische Präparate
○ Andere (weiter unten erwähnen)
○ Keine rezeptfreie pharmakologische oder homöopathische Behandlungen

UND/ODER

Nicht-pharmakologische Behandlung
○ Schlafhygiene
○ Bettzeit-/Schlafrestriktion
○ Kognitive Verhaltenstherapie für Insomnie (KVT-I/CBT-I)
○ Hypnose
○ Entspannungstechniken
○ Andere Psychotherapie
○ Andere (weiter unten erwähnen)
○ Keine nicht-pharmakologische Behandlung
Sonstige ERSTE BEHANDLUNGSWAHL: ____

2. Klinische Vignette - Klinische Vignette 2B

Herr G. ist ein 79-jähriger Mann, der sich in einer ähnlichen Situation wie Frau C. in der Klinischen Vignette 2A befindet, er wacht mehrmals in der Nacht auf und hat Probleme beim Einschlafen. Aufgrund seiner Schlafprobleme ist er tagsüber oft müde und kann nicht alle Aufgaben erledigen, die er erledigen wollte. Er erhält die gleiche medizinische Behandlung (Zolpidem jede Nacht für 3 Monate) wie Frau C., um seinen Schlaf zu verbessern.

Welche rezeptfreien pharmakologischen oder homöopathischen Behandlungen (inkl. Medikamente der Liste B-/B+) könnten Sie sich für diesen Kunden vorstellen zusätzlich oder als Alternative zur Zolpidem-Behandlung?
Kreuzen Sie das Kästchen an, das Ihnen entspricht

	0 (Trifft überhaupt nicht zu)	1 (Trifft nicht zu)	2 (Trifft eher nicht zu)	3 (Trifft eher zu)	4 (Trifft oft zu)	5 (Trifft ganz zu)	Weiß nicht/Keine Antwort
Antihistaminika (z.B. Diphenhydramin (Benocten®) oder Doxylamin (Sanalepsi® N))	○	○	○	○	○	○	○
Baldrian (z.B. Sedonium®, Somnofor® oder kombiniert mit anderen Phytotherapeutika (Dormiplant®, Redormin®))	○	○	○	○	○	○	○
Andere Phytotherapeutika (z.B. Lavendel (Lasea®), Kräutertee)	○	○	○	○	○	○	○
Homöopathische Präparate	○	○	○	○	○	○	○
Andere Behandlungen (weiter unten erwähnen)	○	○	○	○	○	○	○

Keine der hier genannten Behandlungen	◯	◯	◯	◯	◯	◯	◯

Andere Behandlungen, die Sie sich für diesen Kunden vorstellen könnten:

____

Welche verschreibungspflichtigen Medikamente würden Sie dem Arzt bzw. der Ärztin in einer interprofessionellen Diskussion empfehlen als Alternative zu Zolpidem?

Kreuzen Sie das Kästchen an, das Ihnen entspricht

	0 (Trifft überhaupt nicht zu)	1 (Trifft nicht zu)	2 (Trifft eher nicht zu)	3 (Trifft eher zu)	4 (Trifft oft zu)	5 (Trifft ganz zu)	Weiß nicht/Keine Antwort
Keine Alternative: Z-Substanzen beibehalten (z.B. Zolpidem (Stilnox®), Zopiclon (Imovane®))	◯	◯	◯	◯	◯	◯	◯
Benzodiazepine (z.B. Lorazepam (Seresta®))	◯	◯	◯	◯	◯	◯	◯
Antidepressiva mit sedierender Wirkung (z.B. Mirtazapin (Remeron®), Trazodon (Trittico®))	◯	◯	◯	◯	◯	◯	◯
Neuroleptika (z.B. Quetiapin (Sequase®, Seroquel®))	◯	◯	◯	◯	◯	◯	◯
Melatonin (z.B. Circadin®)	◯	◯	◯	◯	◯	◯	◯
Andere verschreibungspflichtige Medikamente (weiter unten erwähnen)	◯	◯	◯	◯	◯	◯	◯
Keine Empfehlung von verschreibungspflichtigen Medikamenten	◯	◯	◯	◯	◯	◯	◯

Andere verschreibungspflichtige Medikamente, die Sie dem Arzt*in für diese Kundin empfehlen würden:

____

Welche nicht-pharmakologischen Alternativbehandlungen oder zusätzlich zu Zolpidem könnten Sie sich für diesen Kunden vorstellen?

Kreuzen Sie das Kästchen an, das Ihnen entspricht

	0 (Trifft überhaupt nicht zu)	1 (Trifft nicht zu)	2 (Trifft eher nicht zu)	3 (Trifft eher zu)	4 (Trifft oft zu)	5 (Trifft ganz zu)	Weiß nicht/Keine Antwort
Schlafhygiene	◯	◯	◯	◯	◯	◯	◯
Bettzeit-/Schlafrestriktion	◯	◯	◯	◯	◯	◯	◯
Kognitive Verhaltenstherapie für Insomnie (KVT-I/CBT-I)	◯	◯	◯	◯	◯	◯	◯
Hypnose	◯	◯	◯	◯	◯	◯	◯
Entspannungstechniken	◯	◯	◯	◯	◯	◯	◯
Andere Psychotherapie	◯	◯	◯	◯	◯	◯	◯
Andere nicht-pharmakologische Behandlungen (weiter unten erwähnen)	◯	◯	◯	◯	◯	◯	◯
Keine der hier genannten nicht-pharmakologische Behandlung	◯	◯	◯	◯	◯	◯	◯

Andere nicht-pharmakologische Behandlungen, die Sie sich für diesen Kunden vorstellen könnten:

____

Unter den oben genannten Optionen, was wäre für Sie die ERSTE BEHANDLUNGSWAHL (pharmakologische (inkl. Liste B+/B-), homöopathische und/oder nicht pharmakologische) für diesen Kunden in der Apotheke?

Sie können eine ***Behandlung erster Wahl*** *für eine pharmakologische* ***ODER*** *nicht-pharmakologische Behandlung* ***ODER*** *beides angeben.*

Rezeptfreie pharmakologische oder homöopathische Behandlungen

◯ Antihistaminika (z.B. Diphenhydramin (Benocten®) oder Doxylamin (Sanalepsi® N))
◯ Baldrian (z.B. Sedonium®, Somnofor® oder kombiniert mit anderen Phytotherapeutika (Dormiplant®, Redormin®))
◯ Andere Phytotherapeutika (z.B. Lavendel (Lasea®), Kräutertee)
◯ Homöopathische Präparate
◯ Andere (weiter unten erwähnen)
◯ Keine rezeptfreie pharmakologischen oder homöopathischen Behandlungen

UND/ODER

Nicht-pharmakologische Behandlung

- ○ Schlafhygiene
- ○ Bettzeit-/Schlafrestriktion
- ○ Kognitive Verhaltenstherapie für Insomnie (KVT-I/CBT-I)
- ○ Hypnose
- ○ Entspannungstechniken
- ○ Andere Psychotherapie
- ○ Andere (weiter unten erwähnen)
- ○ Keine nicht-pharmakologische Behandlung

Sonstige ERSTE BEHANDLUNGSWAHL: ____

3. Allgemeine Wahrnehmung von Insomnie

Wie hoch ist Ihrer Meinung nach der Anteil der Kund*innen, die unter Schlafstörungen leiden?

Hinsichtlich jeglicher Ursache

Subjektive Bewertung

- ○ <5%
- ○ 5%-15%
- ○ 15%-30%
- ○ 30-50%
- ○ 50%-75%
- ○ >75%
- ○ Weiß nicht/Keine Antwort

Wie hoch ist Ihrer Meinung nach der Anteil der Kund*innen, die unter akuter Insomnie leiden (3 oder mehr Nächte pro Woche mit Insomnie für WENIGER als 3 Monate)?

Akute Insomnie wurde nicht unbedingt vorher diagnostiziert

Subjektive Einschätzung des Verhältnisses

- ○ <5%
- ○ 5%-15%
- ○ 15%-30%
- ○ 30-50%
- ○ 50%-75%
- ○ >75%
- ○ Weiß nicht/Keine Antwort

Wie hoch ist Ihrer Meinung nach der Anteil der Kund*innen, die unter chronische Insomnie leiden (3 oder mehr Nächte pro Woche mit Insomnie für MEHR als 3 Monate)?

Chronische Insomnie wurde vorher nicht unbedingt diagnostiziert

Subjektive Einschätzung des Verhältnisses

- ○ <5%
- ○ 5%-15%
- ○ 15%-30%
- ○ 30-50%
- ○ 50%-75%
- ○ >75%
- ○ Weiß nicht/Keine Antwort

Wie hoch ist Ihrer Meinung nach der Anteil der Kund*innen, die regelmäßig und langfristig Benzodiazepine oder die Z-Substanz einnehmen (>1x/Woche für >6 Monate)?

Alle verschreibungspflichtigen Indikationen zusammen

Subjektive Bewertung

- ○ <5%
- ○ 5%-15%
- ○ 15%-30%
- ○ 30-50%
- ○ 50%-75%
- ○ >75%
- ○ Weiß nicht/Keine Antwort

Wie beurteilen Sie die Wirksamkeit der nachstehenden homöopathischen und pharmakologischen Behandlungen (pharmakologische Behandlungen mit oder ohne ärztliche Verschreibung) zusätzlich zum Placebo-Effekts bei der

Kurzzeitbehandlung (<1 Monat) von chronischer Insomnie (3 oder mehr Nächte pro Woche mit Insomnie für MEHR als 3 Monate)?
Kreuzen Sie das Kästchen an, das Ihnen entspricht

	0 (Überhaupt nicht wirksam)	1 (Nicht wirksam)	2 (Eher nicht wirksam)	3 (Eher wirksam)	4 (Sehr wirksam)	5 (Extrem wirksam)	Weiß nicht/Keine Antwort
Antihistaminika (z.B. Diphenhydramin (Benocten®) oder Doxylamin (Sanalepsi® N))	○	○	○	○	○	○	○
Baldrian (z.B. Sedonium®, Somnofor® oder kombiniert mit anderen Phytotherapeutika (Dormiplant®, Redormin®))	○	○	○	○	○	○	○
Andere Phytotherapeutika (z.B. Lavendel (Lasea®), Kräutertee)	○	○	○	○	○	○	○
Homöopathische Präparate	○	○	○	○	○	○	○
Z-Substanz (z.B. Zolpidem (Stilnox®), Zopiclon (Imovane®))	○	○	○	○	○	○	○
Benzodiazepine (z.B. Lorazepam (Seresta®))	○	○	○	○	○	○	○
Antidepressiva mit sedierender Wirkung (z.B. Mirtazapin (Remeron®), Trazodon (Trittico®))	○	○	○	○	○	○	○
Neuroleptika (z.B. Quetiapin (Sequase®, Seroquel®))	○	○	○	○	○	○	○
Melatonin (z.B. Circadin®)	○	○	○	○	○	○	○

Wie häufig empfehlen Sie Ihren Kund*innen die folgenden Elemente der Schlafhygiene?

	Immer oder fast immer (in ≥90 % der Gespräche)	Sehr oft (in ≥50% - <90% der Gespräche)	Häufig (in ≥10% - <50% der Gespräche)	Selten oder nie (in <10% der Gespräche)	Weiß nicht/Keine Antwort
Kein Kaffee, kein Tee und kein Kakao nach 16:00 Uhr	○	○	○	○	○
Leichtes Abendessen	○	○	○	○	○
Körperliche Bewegung tagsüber, aber nicht abends	○	○	○	○	○
Keine Bildschirmzeit direkt vor dem Schlafengehen	○	○	○	○	○
Ritual beim Schlafengehen einführen	○	○	○	○	○
Kühlen die Temperatur des Schlafzimmers	○	○	○	○	○
Entspannungsmethode	○	○	○	○	○
Lichttherapie	○	○	○	○	○
Bei Insomnie nicht im Bett bleiben	○	○	○	○	○
Bettzeit-/Schlafrestriktion	○	○	○	○	○
Aufwachen immer zur gleichen Zeit	○	○	○	○	○
Nickerchen tagsüber vermeiden	○	○	○	○	○

Andere: ____

Wie viel Zeit brauchen Sie im Durchschnitt für die Gespräch (Anamnese und Behandlungsmöglichkeiten) von Schlafstörungen?

○ <5 Min
○ 5-10 Min

◯ 10-15 Min
◯ >15 Min
◯ Weiß nicht/Keine Antwort

Wie viel Zeit brauchen Sie im Durchschnitt um über Schlafhygiene zu beraten?
◯ <5 Min
◯ 5-10 Min
◯ 10-15 Min
◯ >15 Min
◯ Weiß nicht/Keine Antwort

Bitte denken Sie an Ihre letzten 10 Kund*innen, die Sie wegen chronischer Insomnie beraten haben. Wie viele ihrer Beratungen haben Sie in Ihrem Beratungsraum vorgenommen?
◯ Keine
◯ 1-2 mal
◯ 3-5 mal
◯ 6-7 mal
◯ 8-9 mal
◯ Alle
◯ Weiß nicht/Keine Antwort

Haben Sie in Ihrer Apotheke einen Algorithmus zur Behandlung von Kund*innen mit Schlafstörungen oder chronischer Insomnie?
◯ Ja
◯ Nein
◯ Weiß nicht/Keine Antwort

Wir wären Ihnen sehr dankbar, wenn Sie den Algorithmus hochladen oder an die folgende E-Mail Adresse senden könnten: fanny.mulder@students.unibe.ch
Die Adresse ist auch in der E-Mail zu finden.

Wären Sie daran interessiert, einen Algorithmus zur Behandlung von Schlafstörungen und chronischer Insomnie zu erhalten?
◯ Ja
◯ Nein
◯ Weiss nicht/Keine Antwort
◯ Kommentare

4. CBT-I Kenntnisse und Erfahrungen

Gemäss Gemäss neuen Leitlinien zur Behandlung der chronischen Insomnie ist die kognitive Verhaltenstherapie für Insomnie (KVT-I oder CBT-I) die Behandlung der ersten Wahl[1]. CBT-I ist eine Verhaltenstherapie, die Psychoedukation, Entspannungstechniken, verschiedene kognitive Methoden, Schlafrestriktion und Stimulus-Kontrolle umfasst. Während CBT-I bisher hauptsächlich von spezialisierten Psychologen angeboten wurde, existiert heutzutage eine alternative Online-Form und könnte einen größeren Teil der Bevölkerung erreichen.
[1]i.m@ail-offizin, Pflanzliche Sedativa und Hypnotika, Nr. 11 / 15.06.2020

Bevor Sie diesen Fragebogen ausgefüllt haben, haben Sie schon einmal von CBT-I gehört?
◯ Ja
◯ Nein
◯ Weiss nicht/Keine Antwort
◯ Kommentare

Wenn Sie bereits von CBT-I gehört haben, haben Sie Ihren Kund*innen jemals CBT-I empfohlen?
◯ Nie
◯ <1x/Monat
◯ 1x/Monat bis <1x/Woche
◯ 1x/Woche oder mehr
◯ Weiß nicht/Keine Antwort

K**ennen Sie ein*e Psycholog*in oder CBT-I-Spezialist*in, an die Sie Ihren Kund*innen empfohlen könnten?**
◯ Ja
◯ Nein
◯ Weiss nicht/Will nicht antworten

Haben Sie in der Vergangenheit Kund*innen empfohlen ein*e Psycholog*in oder CBT-I-Spezialist*in aufzusuchen?

○ Nie
○ <1x/Monat
○ 1x/Monat bis <1x/Woche
○ 1x/Woche oder mehr
○ Weiß nicht/Will nicht antworten

Kennen Sie eine CBT-I-Website/Applikation, an die Sie Ihren Kund*innen empfohlen könnten?
○ Ja
○ Nein
○ Weiß nicht/Will nicht antworten

Haben Sie in der Vergangenheit Kund*innen zu einer CBT-I-Website/Applikation beraten?
○ Nie
○ <1x/Monat
○ 1x/Monat bis <1x/Woche
○ 1x/Woche oder mehr
○ Weiß nicht/Keine Antwort

Wie groß ist Ihr Interesse daran, zukünftig Kund*innen nach einer CBT-I-Therapie über mehrere Wochen online zu begleiten um ihnen zu helfen, die Therapie abzuschließen?
○ Keine
○ Ein wenig
○ Mittel
○ Viel
○ Sehr viel
○ Weiss nicht/Keine Antwort
○ Kommentare/Erinnerungen/Ideen

Wie groß ist Ihr Interesse an weiteren Schulungen/Kursen zum Thema Schlafstörungen und CBT-I?
○ Keine
○ Ein wenig
○ Mittel
○ Viel
○ Sehr viel
○ Weiss nicht/Keine Antwort
○ Kommentare/Erinnerungen/Ideen

Was bräuchten Sie Ihrer Meinung nach noch, um in Ihrer Apotheke eine gute Insomnie Behandlung für Ihre Kund*innen umzusetzen?
☐ Mehr Wissen/Kurse zum Thema
☐ Mehr Platz, um ein persönliches Gespräch mit Kund*innen zu führen (z.B. separates Behandlungszimmer)
☐ Die Möglichkeit die Dienstleistung der Insomnie Beratung (z.B. CBT-I) zu verrechnen
☐ Besserer Kontakt mit Ärzten und Ärztinnen
☐ Weiß nicht/Keine Antwort
☐ Andere

Sonstige Fragen/Kommentare/Vorschläge/Ideen zur Behandlung von Schlafstörungen
____

Supplementary Materials S4: Online questionnaire, French online version

Questionnaire : Variations dans les conseils pour client.es souffrant d'insomnie chronique dans les pharmacies suisses : une étude d'enquête basée sur des vignettes cliniques

Texte d'introduction

Chère et cher collègue,
Par le biais de ce questionnaire, nous visons à décrire comment les pharmacien.ne.s en Suisse conseillent et traitent des client.e.s adultes (>18 ans) souffrant d'insomnie chronique. Dans cette optique, nous présentons trois vignettes cliniques, par lesquelles nous aimerions comprendre votre pratique clinique actuelle. Les vignettes cliniques sont suivies de questions générales au sujet de votre perception de la prévalence de l'insomnie chronique et plus spécifiquement au sujet de la thérapie cognitive-comportementale (TCC-I, en anglais : cognitive-behavioral therapy for insomnia (CBT-I)) pour le traitement de l'insomnie chronique. Nous sommes également intéressé.e.s à vos suggestions qui pourraient améliorer le traitement des client.e.s souffrant d'insomnie chronique. A la fin de l'enquête, vous trouverez un champ pour vos commentaires que nous vous encourageons à remplir.
Le but du questionnaire n'est pas de tester vos connaissances, mais surtout de décrire comment les pratiques cliniques peuvent parfois différer d'une pharmacie à l'autre. La pratique clinique est complexe et souvent, il n'y a pas une approche juste ou fausse. Comme vous le savez, les recommandations de pratique clinique différent également. Ce travail permettra de développer des interventions futures sur la base de la pratique clinique actuelle.
Le questionnaire a été développé et testé de manière interprofessionnelle avec des pharmacien.ne.s et des médecins de premier recours et spécialistes.
Selon notre expérience issue des tests préliminaire, il faut compter environ 25 minutes pour compléter le questionnaire. Toutes vos réponses seront collectées de manière anonyme. Les données seront analysées et feront l'objet d'une publication dans une revue à politique éditoriale, que nous disséminerons après publication. Nous vous remercions de votre participation.

1. Informations générales - a. Données personnelles

Sexe
○ Homme
○ Femme
○ Autre

Tranche d'âge
○ <30
○ 31-40
○ 41-50
○ 51-60
○ >60

Depuis combien de temps travaillez-vous comme pharmacien.ne d'officine ?
○ <5ans
○ 6-15ans
○ 16-25ans
○ 26-35ans
○ >35ans

Quelle est votre fonction dans l'officine ?
○ Pharmacien.ne propriétaire
○ Gérant.e
○ Adjoint.e
○ Remplaçant.e
○ Autre: ____________________

Dans qu'elle université avez-vous terminé votre master resp. votre diplôme en pharmacie ?
○ ETH Zürich
○ Université de Bâle
○ Université de Genève et/ou de Lausanne
○ Université de Berne
○ Autre université : ____________________

Avez-vous fait des formations supplémentaires ?
☐ Certificat de formation complémentaire FPH « Anamnèse en soins primaires »
☐ Cours de cercle de qualité
☐ CAS « Pharmacie clinique : prestations dans les soins de base » à l'université de Genève

☐ CAS « Klinische Pharmazie » (à l'université de Bâle ou à l'ETH)
☐ Diplôme FPH en pharmacie d'officine
☐ Diplôme FPH en pharmacie d'hôpital
☐ Diplôme FPH en pharmacie clinique
☐ Doctorat
☐ Aucune
☐ Autre: ____

1. Informations générales
b. Données de la pharmacie

Lieu de pratique
◯ Zone rurale
◯ Agglomération
◯ Ville

Dans quel canton se trouve votre pharmacie ?
◯ Appenzell Rh.-Ext.
◯ Appenzell Rh.-Int.
◯ Argovie
◯ Bâle-Campagne
◯ Bâle-Ville
◯ Berne
◯ Fribourg
◯ Genève
◯ Glaris
◯ Grisons
◯ Jura
◯ Lucerne
◯ Neuchâtel
◯ Nidwald
◯ Obwald
◯ Saint-Gall
◯ Schaffhouse
◯ Schwyz
◯ Soleure
◯ Tessin
◯ Thurgovie
◯ Uri
◯ Vaud
◯ Valais
◯ Zoug
◯ Zurich

À quelle forme de remise de médicaments est soumis votre zone géographique de vente ?
◯ Remise uniquement en pharmacie (Rx)
◯ Dispensation médicale (DM)
◯ Forme mixte (FM)

Structure de la pharmacie
◯ Chaîne
◯ Groupe
◯ Pharmacies formant des communautés d'achats
◯ Pharmacie indépendante
◯ Autre : ____

Nombre de collaborateurs en équivalent temps plein (ETP)
(Exemple a : 1 pharmacien 90% + 1 pharmacienne 60% = 150 % d'emplois = 1.5 ETP)
Nombre de pharmacien.ne.s (incluant les étudiant.e.s en année d'assistanat) : ____________ETP
(Exemple b : 1 assistante en pharmacie 80% + 1 assistant en pharmacie CFC 100% + 1 Droguiste 70% = 250 % d'emplois = 2.5 ETP)
Nombre de collaborateurs.trices participant à la vente de médicaments (à l'exclusion des pharmacien.nes): __________ETP

2. Vignette clinique - Vignette clinique 1

Mme R. est une secrétaire de 50 ans dans une entreprise internationale. Il y a quelques mois, son entreprise a dû se résoudre à licencier des personnes et Mme R. a subi un fort stress qui l'a empêchée de dormir durant plusieurs mois. La situation est maintenant stabilisée et votre cliente a l'assurance de maintenir son emploi. Cependant, son sommeil ne s'est pas amélioré. Elle a du mal à s'endormir depuis maintenant près de 4 mois. Par ailleurs, elle se réveille régulièrement durant la nuit et n'arrive ensuite plus à se rendormir. Son manque de sommeil affecte son travail durant la journée, elle se sent moins concentrée et a de la peine à finir le travail qu'elle a à faire dans les temps. Elle vient dans votre pharmacie, car elle aimerait un médicament qui la soulagerait de ces troubles du sommeil

Quels sont les traitements pharmacologiques ou homéopathiques sans ordonnance (incluant les médicaments de la liste B-/B+) que vous envisagez de délivrer à cette cliente ?
Cochez la case qui vous correspond

	0 (Jamais envisagé)	1 (Rarement envisagé)	2 (Peu envisagé)	3 (Parfois envisagé)	4 (Souvent envisagé)	5 (Toujours envisagé)	Ne sais pas/Pas de réponse
Antihistaminique (par ex : Diphenhydramin (Benocten®) ou Doxylamin (Sanalepsi® N))	○	○	○	○	○	○	○
Valériane (par ex : Sedonium®, Somnofor® ou mixte avec autre phytothérapie (Dormiplant®, Redormin®))	○	○	○	○	○	○	○
Autre phytothérapie (par ex : Lavande (Lasea®), tisanes)	○	○	○	○	○	○	○
Traitement homéopathique	○	○	○	○	○	○	○
Autres traitements (noter ci-dessous)	○	○	○	○	○	○	○
Aucun des traitements ci-dessus	○	○	○	○	○	○	○

Autres traitements que vous conseillez :
____

Quels sont les médicaments sur ordonnance que vous recommanderiez à un.e médecin lors d'un échange interprofessionnel pour cette cliente ?
Cochez la case qui vous correspond

	0 (Jamais envisagé)	1 (Rarement envisagé)	2 (Peu envisagé)	3 (Parfois envisagé)	4 (Souvent envisagé)	5 (Toujours envisagé)	Ne sais pas/Pas de réponse
Substance Z (par ex : Zolpidem (Stilnox®), Zopiclon (Imovane®))	○	○	○	○	○	○	○
Benzodiazépine (par ex : Lorazepam (Seresta®))	○	○	○	○	○	○	○
Antidépresseur avec effet sédatif (par ex : Mitrazapine (Remeron®) Trazodone (Trittico®))	○	○	○	○	○	○	○
Neuroleptique (par ex : Quetiapine (Sequase®, Seroquel®))	○	○	○	○	○	○	○
Mélatonine (par ex : Circadin®)	○	○	○	○	○	○	○
Autres médicaments sur ordonnance (noter ci-dessous)	○	○	○	○	○	○	○
Un autre traitement, mais pas de médicament sur ordonnance	○	○	○	○	○	○	○

Autres médicaments sur ordonnance que vous recommanderiez pour cette cliente : ______________________________________

Quels sont les traitements non-pharmacologiques que vous envisagez pour cette cliente ?
Cochez la case qui vous correspond

	0 (Jamais envisagé)	1 (Rarement envisagé)	2 (Peu envisagé)	3 (Parfois envisagé)	4 (Souvent envisagé)	5 (Toujours envisagé)	Ne sais pas/Pas de réponse

Hygiène du sommeil	◯	◯	◯	◯	◯	◯	◯
Restriction du sommeil/diminuer le temps total passé au lit	◯	◯	◯	◯	◯	◯	◯
Thérapie cognitivo-comportementale de l'insomnie (TCC-I ou CBT-I)	◯	◯	◯	◯	◯	◯	◯
Hypnose	◯	◯	◯	◯	◯	◯	◯
Thérapie de relaxation	◯	◯	◯	◯	◯	◯	◯
Autre psychothérapie	◯	◯	◯	◯	◯	◯	◯
Autres traitements non-pharmacologiques (noter ci-dessous)	◯	◯	◯	◯	◯	◯	◯
Pas de traitement non-pharmacologique	◯	◯	◯	◯	◯	◯	◯

Autres traitements non-pharmacologiques que vous envisagez pour cette cliente : ____

Parmi les options ci-dessous, quel serait pour vous le traitement pharmacologique ou homéopathique sans ordonnance (incluant les médicaments de la liste B-/B+) et non-pharmacologique DE PREMIER CHOIX en pharmacie pour cette cliente ?
*Vous pouvez indiquer un **traitement de premier choix** pour traitement pharmacologique **OU** non-pharmacologique **OU** les deux.*
Traitement pharmacologique ou homéopathique sans ordonnance :
◯ Antihistaminique (par ex : Diphenhydramin (Benocten®) ou Doxylamin (Sanalepsi® N))
◯ Valériane (par ex : Sedonium®, Somnofor® ou mixte avec autre phytothérapie (Dormiplant®, Redormin®))
◯ Autre phytothérapie (par ex : Lavande (Lasea®), tisanes)
◯ Traitement homéopathique
◯ Autre (noter ci-dessous)
◯ Pas de traitement pharmacologique ou homéopathique sans ordonnance

ET/OU

Traitement non-pharmacologique :
◯ Hygiène du sommeil
◯ Restriction du sommeil/diminuer le temps total passé au lit
◯ Thérapie cognitivo-comportementale de l'insomnie (TCC-I ou CBT-I)
◯ Hypnose
◯ Thérapie de relaxation
◯ Autre psychothérapie
◯ Autre (noter ci-dessous)
◯ Pas de traitement non-pharmacologique
Autre TRAITEMENT DE PREMIER CHOIX: ____

Dans une situation telle que celle-ci, quels sont les éléments dont vous allez discuter avec votre client.e ?
Cochez la case qui vous correspond.

	Toujours ou presque (≥90% des fois)	Très souvent (≥50% - <90% des fois)	Souvent (≥10% - <50% des fois)	Rarement ou jamais (<10% des fois)	Ne sais pas/Pas de réponse
Remise de traitements (pharmacologique (incluant B+/B-) ou homéopathique sans ordonnance) sans anamnèse supplémentaire	◯	◯	◯	◯	◯
Anamnèse du sommeil	◯	◯	◯	◯	◯
Anamnèse médicamenteuse	◯	◯	◯	◯	◯
Revue des comorbidité	◯	◯	◯	◯	◯
Histoire personnelle	◯	◯	◯	◯	◯
Anamnèse dans salle de consultation dédiée (au lieu d'anamnèse au comptoir)	◯	◯	◯	◯	◯

Autres éléments de discussion: ____

2. Vignette clinique- Vignette clinique 2A

Mme C. est une caissière de 45 ans ayant trois enfants. Elle a divorcé il y a moins d'un an et a désormais des problèmes à boucler les fins de mois. Elle se réveille régulièrement durant la nuit et également plus tôt le matin sans pouvoir se rendormir. Elle se sent tout le temps fatigué et a du mal à s'occuper de ses enfants quand elle rentre à la maison le soir. Elle est allée voir sa médecin il y a quelques mois et prend du Zolpidem chaque soir depuis trois mois. La médecin a renouvelé l'ordonnance de Zolpidem pour trois mois de plus. Cette cliente, que vous voyez pour la première fois, vient retirer ce médicament dans votre pharmacie.

Quels sont les traitements pharmacologiques ou homéopathiques sans ordonnance (incluant médicaments de la liste B-/B+) alternatifs ou complémentaires au zolpidem que vous envisagez de délivrer à cette cliente ?

Cochez la case qui vous correspond

	0 (Jamais envisagé)	1 (Rarement envisagé)	2 (Peu envisagé)	3 (Parfois envisagé)	4 (Souvent envisagé)	5 (Toujours envisagé)	Ne sais pas/Pas de réponse
Antihistaminique (par ex : Diphenhydramin (Benocten®) ou Doxylamin (Sanalepsi® N))	○	○	○	○	○	○	○
Valériane (par ex : Sedonium®, Somnofor® ou mixte avec autre phytothérapie (Dormiplant®, Redormin®))	○	○	○	○	○	○	○
Autre phytothérapie (par ex : Lavande (Lasea®), tisanes)	○	○	○	○	○	○	○
Traitement homéopathique	○	○	○	○	○	○	○
Autres traitements (noter ci-dessous)	○	○	○	○	○	○	○
Aucun des traitements ci-dessus	○	○	○	○	○	○	○

Autres traitements que vous conseillez: ________________________________

Quels sont les médicaments sur ordonnance alternatifs au Zolpidem que vous recommanderiez à un.e médecin lors d'un échange interprofessionnel pour cette cliente ?

Cochez la case qui vous correspond

	0 (Jamais envisagé)	1 (Rarement envisagé)	2 (Peu envisagé)	3 (Parfois envisagé)	4 (Souvent envisagé)	5 (Toujours envisagé)	Ne sais pas/Pas de réponse
Pas d'alternative: Substance Z (par ex: Zolpidem (Stilnox®), Zopiclon (Imovane®))	○	○	○	○	○	○	○
Benzodiazépine (par ex : Lorazepam (Seresta®))	○	○	○	○	○	○	○
Antidépresseur avec effet sédatif (par ex : Mitrazapine (Remeron®) Trazodone (Trittico®))	○	○	○	○	○	○	○
Neuroleptique (par ex : Quetiapine (Sequase®, Seroquel®))	○	○	○	○	○	○	○
Mélatonine (par ex : Circadin®)	○	○	○	○	○	○	○
Autres médicaments sur ordonnance (noter ci-dessous)	○	○	○	○	○	○	○
Pas de médicament sur ordonnance	○	○	○	○	○	○	○

Autres médicaments sur ordonnance que vous recommanderiez pour cette cliente : ____________________

Quels sont les traitements non-pharmacologiques alternatifs ou complémentaires au zolpidem que vous envisagez pour cette cliente ?

Cochez la case qui vous correspond

	0 (Jamais envisagé)	1 (Rarement envisagé)	2 (Peu envisagé)	3 (Parfois envisagé)	4 (Souvent envisagé)	5 (Toujours envisagé)	Ne sais pas/Pas de réponse
Hygiène du sommeil	○	○	○	○	○	○	○

Restriction du sommeil/diminuer le temps total passé au lit	○	○	○	○	○	○	○
Thérapie cognitivo-comportementale de l'insomnie (TCC-I ou CBT-I)	○	○	○	○	○	○	○
Hypnose	○	○	○	○	○	○	○
Thérapie de relaxation	○	○	○	○	○	○	○
Autre psychothérapie	○	○	○	○	○	○	○
Autres traitements non-pharmacologiques (noter ci-dessous)	○	○	○	○	○	○	○
Pas de traitement non-pharmacologique	○	○	○	○	○	○	○

Autres traitements non-pharmacologiques que vous envisagez pour cette cliente : ____

Parmi les options ci-dessous, quel serait pour vous le traitement pharmacologique ou homéopathique sans ordonnance (incluant médicaments de la liste B-/B+) et non-pharmacologique DE PREMIER CHOIX en pharmacie pour cette cliente ?
*Vous pouvez indiquer un **traitement de premier choix** pour traitement pharmacologique **OU** non-pharmacologique **OU** les deux.*
Traitement pharmacologique ou homéopathique sans ordonnance
○ Antihistaminique (par ex : Diphenhydramin (Benocten®) ou Doxylamin (Sanalepsi® N))
○ Valériane (par ex : Sedonium®, Somnofor® ou mixte avec autre phytothérapie (Dormiplant®, Redormin®))
○ Autre phytothérapie (par ex : Lavande (Lasea®), tisanes)
○ Traitement homéopathique
○ Autre (noter ci-dessous)
○ Pas de traitement pharmacologique ou homéopathique sans ordonnance

ET/OU

Traitement non-pharmacologique
○ Hygiène du sommeil
○ Restriction du sommeil/diminuer le temps total passé au lit
○ Thérapie cognitivo-comportementale de l'insomnie (TCC-I ou CBT-I)
○ Hypnose
○ Thérapie de relaxation
○ Autre psychothérapie
○ Autre (noter ci-dessous)
○ Pas de traitement non-pharmacologique

Autre TRAITEMENT DE PREMIER CHOIX: ____

2. Vignette clinique - Vignette clinique 2B

M. G est un homme de 79 ans qui se trouve dans une situation similaire à Mme C. de la vignette clinique 2A, il se réveille plusieurs fois durant la nuit et a en plus du mal à s'endormir. À cause de ses problèmes de sommeil, il est souvent fatigué durant la journée et n'arrive pas à accomplir toutes les tâches qu'il voulait. Il reçoit le même traitement médical (prise de Zolpidem chaque soir depuis 3 mois) que Mme C. pour améliorer son sommeil.

Quels sont les traitements pharmacologiques ou homéopathiques sans ordonnance (incluant médicaments de la liste B-/B+) alternatifs ou complémentaires au zolpidem que vous envisagez de délivrer à ce client ?
Cochez la case qui vous correspond

	0 (Jamais envisagé)	1 (Rarement envisagé)	2 (Peu envisagé)	3 (Parfois envisagé)	4 (Souvent envisagé)	5 (Toujours envisagé)	Ne sais pas/Pas de réponse
Antihistaminique (par ex : Diphenhydramin (Benocten®) ou Doxylamin (Sanalepsi® N))	○	○	○	○	○	○	○
Valériane (par ex : Sedonium®, Somnofor® ou mixte avec autre phytothérapie (Dormiplant®, Redormin®))	○	○	○	○	○	○	○

Autre phytothérapie (par ex : Lavande (Lasea®), tisanes)	○	○	○	○	○	○	○
Traitement homéopathique	○	○	○	○	○	○	○
Autres traitements (noter ci-dessous)	○	○	○	○	○	○	○
Aucun des traitements ci-dessus	○	○	○	○	○	○	○

Autres traitements que vous conseillez: ____

Quels sont les médicaments sur ordonnance alternatif au Zolpidem que vous recommanderiez à un.e médecin lors d'un échange interprofessionnel pour ce client ?
Cochez la case qui vous correspond

	0 (Jamais envisagé)	1 (Rarement envisagé)	2 (Peu envisagé)	3 (Parfois envisagé)	4 (Souvent envisagé)	5 (Toujours envisagé)	Ne sais pas/Pas de réponse
Substance Z (par ex: Zolpidem (Stilnox®), Zopiclon (Imovane®))	○	○	○	○	○	○	○
Benzodiazépine (par ex : Lorazepam (Seresta®))	○	○	○	○	○	○	○
Antidépresseur avec effet sédatif (par ex : Mitrazapine (Remeron®) Trazodone (Trittico®))	○	○	○	○	○	○	○
Neuroleptique (par ex : Quetiapine (Sequase®, Seroquel®))	○	○	○	○	○	○	○
Mélatonine (par ex : Circadin®)	○	○	○	○	○	○	○
Autres médicaments sur ordonnance (noter ci-dessous)	○	○	○	○	○	○	○
Pas de médicament sur ordonnance	○	○	○	○	○	○	○

Autres médicaments sur ordonnance que vous recommanderiez pour ce client :
____

Quels sont les traitements non-pharmacologiques alternatifs ou complémentaires au zolpidem que vous envisagez pour ce client ?
Cochez la case qui vous correspond

	0 (Jamais envisagé)	1 (Rarement envisagé)	2 (Peu envisagé)	3 (Parfois envisagé)	4 (Souvent envisagé	5 (Toujours envisagé)	Ne sais pas/Pas de réponse
Hygiène du sommeil	○	○	○	○	○	○	○
Restriction du sommeil/ diminuer le temps total passé au lit	○	○	○	○	○	○	○
Thérapie cognitivo-comportementale de l'insomnie (TCC-I ou CBT-I)	○	○	○	○	○	○	○
Hypnose	○	○	○	○	○	○	○
Thérapie de relaxation	○	○	○	○	○	○	○
Autre psychothérapie	○	○	○	○	○	○	○
Autres traitements non-pharmacologiques (noter ci-dessous)	○	○	○	○	○	○	○
Pas de thérapie non-pharmacologique	○	○	○	○	○	○	○

Autres traitements non-pharmacologiques que vous envisagez pour ce client : ____________________________________

Parmi les options ci-dessous, quel serait pour vous le traitement pharmacologique ou homéopathique sans ordonnance (incluant médicaments de la liste B-/B+) et non-pharmacologique DE PREMIER CHOIX en pharmacie pour ce client ?
Vous pouvez indiquer un ***traitement de premier choix*** *pour traitement pharmacologique* ***OU*** *non-pharmacologique* ***OU*** *les deux.*
Traitement pharmacologique ou homéopathique sans ordonnance
- ◯ Antihistaminique (par ex : Diphenhydramin (Benocten®) ou Doxylamin (Sanalepsi® N))
- ◯ Valériane (par ex : Sedonium®, Somnofor® ou mixte avec autre phytothérapie (Dormiplant®, Redormin®))
- ◯ Autre phytothérapie (par ex : Lavande (Lasea®), tisanes)
- ◯ Traitement homéopathique
- ◯ Autre (noter ci-dessous)
- ◯ Pas de traitement pharmacologique ou homéopathique sans ordonnance

ET/OU

Traitement non-pharmacologique
- ◯ Hygiène du sommeil
- ◯ Restriction du sommeil/ diminuer le temps total passé au lit
- ◯ Thérapie cognitivo-comportementale de l'insomnie (TCC-I ou CBT-I)
- ◯ Hypnose
- ◯ Thérapie de relaxation
- ◯ Autre psychothérapie
- ◯ Autre (noter ci-dessous)
- ◯ Pas de traitement non-pharmacologique

Autre TRAITEMENT DE PREMIER CHOIX: ____

3. Perception générale de l'insomnie

Chez quel pourcentage (%) de vos client.e.s estimez-vous la présence de troubles de sommeil (toutes causes et durées confondues)?
Évaluation subjective
- ◯ <5%
- ◯ 5%-15%
- ◯ 15%-30%
- ◯ 30-50%
- ◯ 50%-75%
- ◯ >75%
- ◯ Ne sais pas/Pas de réponse

Selon vous qu'elle est la proportion de vos client.e.s qui souffre d'insomnie aiguë (3 nuits d'insomnie ou plus par semaines depuis MOINS de 3 mois) ?
L'insomnie aiguë peut avoir été diagnostiquée au préalable ou non
Évaluation subjective de la proportion
- ◯ <5%
- ◯ 5%-15%
- ◯ 15%-30%
- ◯ 30-50%
- ◯ 50%-75%
- ◯ >75%
- ◯ Ne sais pas/Pas de réponse

Selon vous qu'elle est la proportion de vos client.e.s qui souffre d'insomnie chronique (3 nuits d'insomnie ou plus par semaines depuis PLUS de 3 mois) ?
L'insomnie chronique peut avoir été diagnostiquée au préalable ou non
Évaluation subjective de la proportion
- ◯ <5%
- ◯ 5%-15%
- ◯ 15%-30%
- ◯ 30-50%
- ◯ 50%-75%
- ◯ >75%
- ◯ Ne sais pas/Pas de réponse

Selon vous qu'elle est la proportion de vos client.e.s qui prend des benzodiazépines ou substances Z régulièrement au long cours ? (>1x/semaine durant >6 mois)
Toutes indications pour la prescription confondue
Évaluation subjective
- ◯ <5%
- ◯ 5%-15%

○ 15%-30%
○ 30-50%
○ 50%-75%
○ >75%
○ Ne sais pas/Pas de réponse

Quelle est votre perception de l'efficacité des traitements homéopathiques et pharmacologiques (traitements pharmacologiques avec ou sans ordonnance médicale) ci-dessous, au-delà de l'effet placebo, pour le traitement à court terme (<1 mois) de l'insomnie chronique (3 nuits d'insomnie ou plus par semaines depuis PLUS de 3 mois) ?
Cochez la case qui vous correspond

	0 (Aucune efficacité)	1 (Très peu d'efficacité)	2 (Peu d'efficacité)	3 (Efficace)	4 (Très efficace)	5 (Extrêmement efficace)	Ne sais pas/Pas de réponse
Antihistaminique (par ex : Diphenhydramin (Benocten®) ou Doxylamin (Sanalepsi® N))	○	○	○	○	○	○	○
Valériane (par ex : Sedonium®, Somnofor® ou mixte avec autre phytothérapie (Dormiplant®, Redormin®))	○	○	○	○	○	○	○
Phytothérapie autre (par ex : Lavande (Lasea®), tisanes)	○	○	○	○	○	○	○
Traitement homéopathique	○	○	○	○	○	○	○
Substance Z (par ex : Zolpidem (Stilnox®), Zopiclon (Imovane®))	○	○	○	○	○	○	○
Benzodiazépine (par ex : Lorazepam (Seresta®))	○	○	○	○	○	○	○
Antidépresseur avec effet sédatif (par ex : Mitrazapine (Remeron®) Trazodone (Trittico®))	○	○	○	○	○	○	○
Neuroleptique (par ex : Quetiapine (Sequase®, Seroquel®))	○	○	○	○	○	○	○
Mélatonine (par ex : Circadin®)	○	○	○	○	○	○	○

Quel sont les différents éléments de l'hygiène de sommeil ci-dessous, que vous conseilleriez à vos client.e.s ?

	Toujours ou presque (≥90% des fois)	Très souvent (≥50 - <90% des fois)	Souvent (≥10 - <50% des fois)	Rarement ou jamais (<10% des fois)	Ne sais pas/Ne veux pas répondre
Pas de café ni de thé ni de cacao après 16h	○	○	○	○	○
Souper léger	○	○	○	○	○
De l'exercice physique durant la journée mais pas le soir	○	○	○	○	○
Pas d'écran juste avant d'aller au lit	○	○	○	○	○
Mettre en place un rituel du coucher	○	○	○	○	○
Refroidir la température de la chambre	○	○	○	○	○
Méthode de relaxation	○	○	○	○	○
Luminothérapie	○	○	○	○	○
Éviter de rester au lit en cas d'insomnie	○	○	○	○	○
Restriction du sommeil/ diminuer le temps total passé au lit	○	○	○	○	○

Se réveiller chaque fois à la même heure	○	○	○	○	○
Éviter les siestes durant la journée	○	○	○	○	○

Autre: ____

Combien de temps accordez-vous en moyenne pour une discussion (anamnèse et possibilités de traitement) concernant les troubles du sommeil ?

○ <5min
○ 5-10min
○ 10-15min
○ >15min
○ Ne sais pas/Pas de réponse

Combien de temps accordez-vous en moyenne pour parler d'hygiène du sommeil ?

○ <5min
○ 5-10min
○ 10-15min
○ >15min
○ Ne sais pas/Pas de réponse

Rappelez-vous des 10 dernier.ère.s client.e.s que vous avez conseillé.e.s concernant les troubles du sommeil, combien de ces conseils avez-vous fait dans votre salle de consultation?

○ Aucun
○ 1-2 fois
○ 3-5 fois
○ 6-7 fois
○ 8-9 fois
○ Tous
○ Ne sais pas/Pas de réponse

Possédez-vous dans votre pharmacie un algorithme pour traiter les personnes ayant des problèmes liés au sommeil ou de l'insomnie chronique ?

○ Oui
○ Non
○ Ne sais pas/Pas de réponse

Nous serions très reconnaissant.es si vous pouviez nous télécharger l'algorithme ci-dessus ou si vous pouviez l'envoyer à l'adresse e-mail suivante: fanny.mulder@students.unibe.ch
L'adresse se trouve également dans le mail.

Seriez-vous intéressé.e à recevoir un algorithme pour traiter les troubles du sommeil et l'insomnie chronique ?

○ Oui
○ Non
○ Ne sais pas/Pas de réponse
○ Commentaire : ____

4. TCC-I/CBT-I: Expérience et connaissance

Selon les nouvelles lignes directrices concernant le traitement de l'insomnie chronique, la thérapie cognitivo-comportementale de l'insomnie (TCC-I ou CBT-I) est le traitement de premier choix[1]. La TTC-I est une thérapie comportementale comprenant de la psychoéducation, des techniques de relaxation, différentes méthodes cognitives, des restrictions de sommeil et des contrôles de stimuli. Alors que la TCC-I était surtout prodiguée par des psychologues spécialisé.e.s, une forme alternative online existe et pourrait permettre de toucher une plus grande partie de la population.

[1] *i.m@il-offizin, Sédatifs et hypnotique à base de plantes, n◦ 12 / 30-06.202043 –*

Avant d'avoir rempli ce questionnaire, aviez-vous déjà entendu parler du TCC-I ?

○ Oui
○ Non
○ Ne sais pas/Pas de réponse
○ Commentaires

Avez-vous déjà recommandé le TCC-I à vos client.e.s ?

○ Jamais
○ <1x/mois

- ◯ 1x/mois à <1x/Semaine
- ◯ 1x/semaine ou plus
- ◯ Ne sais pas/Pas de réponse

Connaissez-vous un.e psychologue ou un.e spécialiste du TCC-I à qui vous pourriez référer vos client.es ?

- ◯ Oui
- ◯ Non
- ◯ Ne sais pas/Pas de réponses

Avez-vous déjà conseillé un.e client.e de s'adresser à un.e psychologue ou un.e spécialiste du TCC-I ?

- ◯ Jamais
- ◯ <1x/mois
- ◯ 1x/mois à <1x/Semaine
- ◯ 1x/semaine ou plus
- ◯ Ne sais pas/Pas de réponse

Connaissez-vous un site/application du TCC-I que vous pourriez référer à vos client.es ?

- ◯ Oui
- ◯ Non
- ◯ Ne sais pas/pas de réponse

Avez-vous déjà conseillé un.e client.e un site/application du TCC-I ?

- ◯ Jamais
- ◯ <1x/mois
- ◯ 1x/mois à <1x/Semaine
- ◯ 1x/semaine ou plus
- ◯ Ne sais pas/Pas de réponse

Quel est votre intérêt dans le futur à suivre sur plusieurs semaines un.e client.e suivant une thérapie TCC-I en ligne, de manière à l'aider à suivre sa thérapie au complet ?

- ◯ Aucun
- ◯ Un peu
- ◯ Moyen
- ◯ Beaucoup
- ◯ Énormément
- ◯ Ne sais pas/Pas de réponse
- ◯ Commentaires/Remarques/Idées

Quel est votre intérêt à suivre des formations/cours concernant les troubles de sommeil et le TCC-I ?

- ◯ Aucun
- ◯ Un peu
- ◯ Moyen
- ◯ Beaucoup
- ◯ Énormément
- ◯ Ne sais pas/Pas de réponse
- ◯ Commentaires/Remarques/Idées: ____

À votre avis, qu'est-ce qu'il vous faudrait en plus pour pouvoir implémenter dans votre pharmacie un bon traitement de l'insomnie pour chancun.e.s de vos client.e.s ?

- ☐ Plus de connaissance/de cours sur la matière
- ☐ Plus d'espace pour avoir une discussion privée avec votre client.e.s (p.e. salle de consultation dédiée)
- ☐ La possibilité de facturer le service de consultation de l'insomnie (par ex: CBT-I)
- ☐ Un meilleur contact avec les médecins
- ☐ Ne sais pas/Pas de réponse
- ☐ Autre

Autres questions/Commentaires/Suggestions/Idées concernant le traitement des troubles du sommeil Pharmacie :

____
